# The Crohn’s disease-associated *Escherichia coli* strain LF82 relies on SOS and stringent responses to survive, multiply and tolerate antibiotics within macrophages

**DOI:** 10.1371/journal.ppat.1008123

**Published:** 2019-11-14

**Authors:** Gaëlle Demarre, Victoria Prudent, Hanna Schenk, Emilie Rousseau, Marie-Agnès Bringer, Nicolas Barnich, Guy Tran Van Nhieu, Sylvie Rimsky, Silvia De Monte, Olivier Espéli

**Affiliations:** 1 CIRB–Collège de France, CNRS-UMR724, INSERM U1050, PSL Research University, Paris, France; 2 Inovarion, Paris, France; 3 Department of Evolutionary Theory, Max Planck Institute for Evolutionary Biology, Plön, Germany; 4 Institut de Biologie de l’Ecole Normale Supérieure, Département de Biologie, Ecole Normale Supérieure, CNRS, INSERM, PSL Research University, Paris, France; 5 Centre des Sciences du Goût et de l'Alimentation, AgroSup Dijon, CNRS, INRA, Université Bourgogne Franche-Comté, Dijon, France; 6 Microbes, Intestin, Inflammation et Susceptibilité de l'Hôte, UMR Inserm/Université Clermont Auvergne U1071, USC INRA 2018, Clermont Ferrand, France; Imperial College London, UNITED KINGDOM

## Abstract

Adherent Invasive *Escherichia coli* (AIEC) strains recovered from Crohn's disease lesions survive and multiply within macrophages. A reference strain for this pathovar, AIEC LF82, forms microcolonies within phagolysosomes, an environment that prevents commensal *E*. *coli* multiplication. Little is known about the LF82 intracellular growth status, and signals leading to macrophage intra-vacuolar multiplication. We used single-cell analysis, genetic dissection and mathematical models to monitor the growth status and cell cycle regulation of intracellular LF82. We found that within macrophages, bacteria may replicate or undergo non-growing phenotypic switches. This switch results from stringent response firing immediately after uptake by macrophages or at later stages, following genotoxic damage and SOS induction during intracellular replication. Importantly, non-growers resist treatment with various antibiotics. Thus, intracellular challenges induce AIEC LF82 phenotypic heterogeneity and non-growing bacteria that could provide a reservoir for antibiotic-tolerant bacteria responsible for relapsing infections.

## Author summary

Intestinal microbiome dysbiosis has been consistently described in patients with Crohn’s disease. The adherent-invasive *E. coli* (AIEC) pathotype in particular, has been implicated in the pathogenesis. AIEC colonize intestinal epithelial cells and survive and replicate within macrophages. Here we demonstrated that growth of AIEC LF82 inside the toxic mature phagolysosomes of macrophages requires sequential adaptations and phenotype switches. First, the stringent response halts the replicative cell cycle of AIEC LF82 for few hours. During this period a large number of AIEC LF82 acquires the ability to tolerate antibiotic treatments. Later when, AIEC multiplication restarts it requires DNA repair suggesting that AIEC have accumulated lesions during the first hours post infection. We observed that non growing bacteria appear frequently from the population of growing AIEC LF82 in this second phase. This raises the proportion of antibiotic tolerant AIEC LF82 up to 10% of the population. Thus, intracellular challenges induce AIEC LF82 phenotypic heterogeneity and non-growing bacteria that could provide a reservoir for antibiotic-tolerant bacteria responsible for relapsing infections.

## Introduction

Adherent Invasive *Escherichia coli* (AIEC) strains recovered from Crohn's disease (CD) lesions are able to adhere to and invade cultured intestinal epithelial cells and to survive and multiply within macrophages [1,2]. Attention around the potential role of AIEC in the pathophysiology of CD is growing [3]; however much remains to be learned about the host-pathogen interactions that govern AIEC infection biology. The diversity of virulence factors displayed by multiple AIEC strains suggests that members of this pathovar have evolved different strategies to colonize their hosts [4]. AIEC ability to persist, and in some cases replicate within macrophages is particularly intriguing. Previous work performed with murine macrophage cell lines has revealed that the prototype AIEC strain LF82, multiplies in a vacuole presenting the characteristics of a mature phagolysosome [5,6]. In such an environment, AIEC should encounter acidic, oxidative, genotoxic and proteic stresses. Screening of genes involved in LF82 fitness within macrophage has revealed that HtrA, DsbA, or Fis proteins are required for optimum fitness, [7–9]. These observations confirmed that LF82 encounter stresses in the phagolysosomes. The impact of these stresses on the survival and growth of LF82 inside phagolysomes has not yet been investigated.

Studies on the bacterial cell cycle of few model organisms under well-controlled laboratory conditions have revealed that to achieve accurate transmission of the genetic information and optimal growth of the population, molecular processes must be coordinated [10,11]. When growth conditions deteriorate, the cell cycle can be modified slightly, as in the case of cell filamentation when genotoxic stress induces the SOS response, or more drastically when sporulation is induced by nutrient deprivation [12]. Such cell cycle alterations affect the entire population. However, under unperturbed conditions, a subset of the population also appears to present a significantly reduced growth rate that allows tolerance to antibiotic treatments. This small portion of the population, typically 1/10000 bacteria, is known as persisters [13–15]. Persisters have been detected for a number of bacteria. They can be found spontaneously in normally growing or stationary phase populations, or they are induced by exogeneous stresses or mutations. Significant increase of the proportion of *S*. *typhimurium* persisters has been observed when these bacteria invade macrophages [16]. Using a fluorescent reporter, it has been demonstrated that these persisters were not multiplying prior to antibiotic addition. Recently, the same tool also revealed the presence of non-growing mycobacteria inside macrophages [17]. Several mediators of persistence have been identified, with toxin-antitoxin modules emerging as key players and the reduction of metabolic activities as the main driver of persistence [18–23]. Persisters are increasingly viewed as a major cause of the recurrence of chronic infectious disease and could be an important factor in the emergence of antibiotic resistance [24]. Tolerance and persistence are similar phenomena of increased survival in the presence of an antibiotic without an increase in the Minimal Inhibitory Concentration (MIC). However, persistence only affects a subpopulation of cells, whereas tolerance is the general ability of a population to survive longer treatments [25]. Tolerant populations survive the period of antibiotic treatment better, with, typically, a weak dependence on the antibiotic concentration. Physiological states favoring tolerance, such as dormancy, reduced metabolism and ATP levels, have also been identified in persistence. Tolerant cells have some aspect of active metabolism, and their frequency in the population changes when bacterial environmental sensing is altered [26–28]. Persistence is often viewed as the result of a phenotypic switch ensuring long-term adaptation to variable environments, however the origin of persistence and tolerance during infection remain unclear, and their distinction in the context of a host-pathogen interactions is difficult [29].

In the present work, we analyzed growth characteristics of the prototype AIEC strain LF82 in THP1 monocyte–derived macrophages. We observed that stresses within macrophages induce a profound bacterial response that leads to the formation of non-growing and antibiotic-tolerant LF82 bacteria at a high rate through the successive induction of stringent and SOS responses. A portion of non-growing LF82 produced within macrophages is tolerant to antibiotics and presents a survival advantage. Our work revealed that internalization within phagolysosomes curbs bacterial multiplication, and frequent escape from the replicative cycle toward non-growing state(s) is a way to improve long-term survival in the host.

## Results

### Inside macrophages, LF82 population size increases despite extensive death

**Fig 1:**
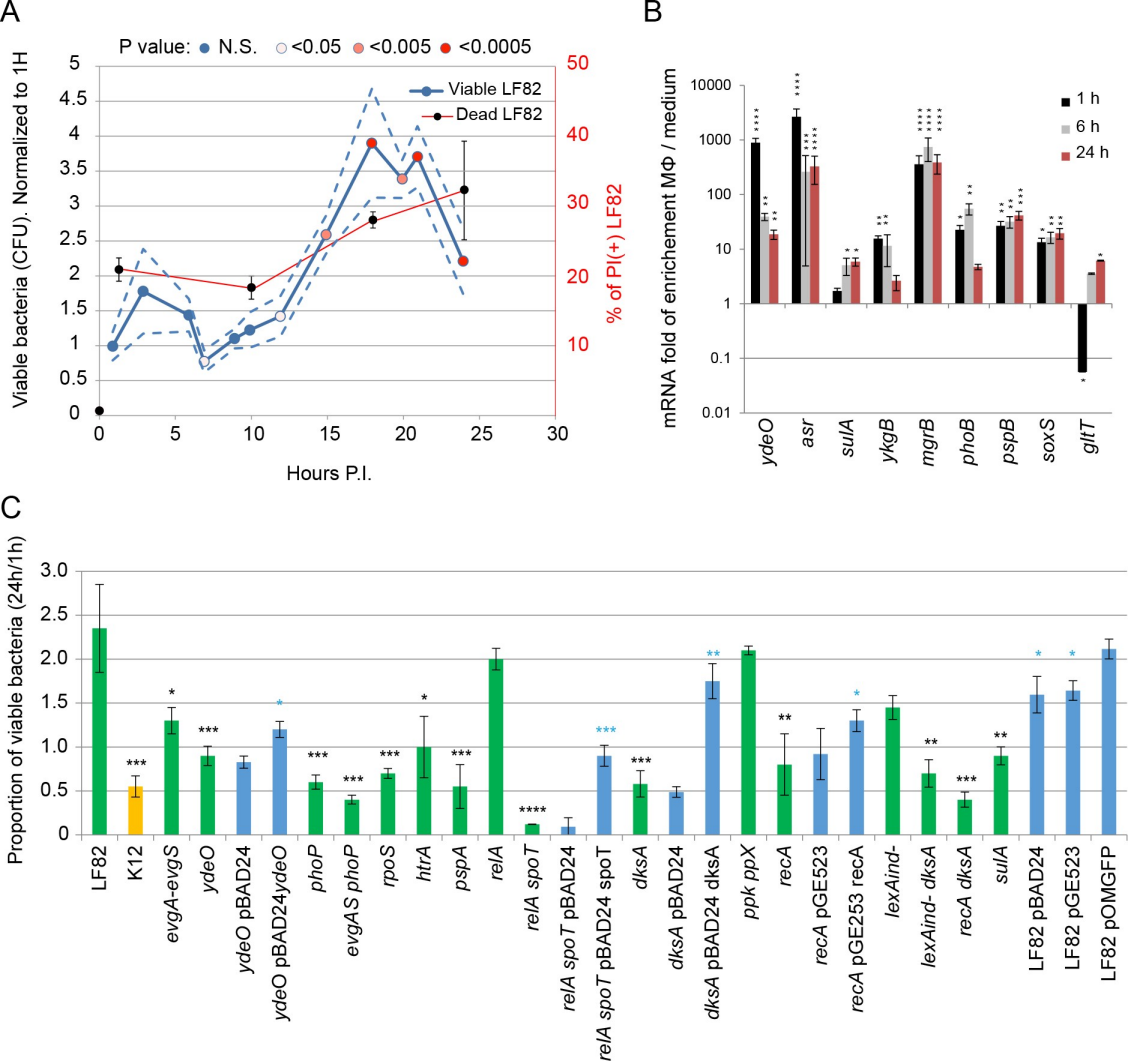
Intracellular LF82 multiplication requires stress responses. A) Measure of viable and dead AIEC LF82 for 24 h post-infection of THP1 differentiated macrophages. Blue circles represent median CFU (blue) ± Standard deviation (SD) (dotted lines). Individual CFU measurements used to compute the curve are presented on S2A Fig. According to the time point considered between 3 and 12 independent infections were performed. Measure of the propidium iodide (PI) positive dead bacteria (black circles) are plotted on the secondary axis B) Analysis by qRT-PCR of the induction of LF82 stress responses at 1 h, 6 h and 24 h P.I. of THP1 macrophages compared to exponential 3 hours of growth (mid exponential) in liquid medium. Values represent the average of four experiments (3 technical replicates each). The uncertainty of the data was analyzed by measuring the number of standard deviations (σ_rep_) separating the average induction of each reporter gene (μ_rep_) with the average induction of the dxr control gene (μ_*dxr*_): * μ_rep_ > μ_dxr_ +/- 3 x σ_rep_; **μ_rep_ > μ_dxr_ + 10 x σ_rep_; ***μ_rep_ > μ_dxr_ +50 x σ_rep_, **** μ_rep_ > μ_dxr_ + 100x σ_rep_. C) Proportion of viable bacteria at 24 h P.I. of THP1 macrophages in comparison to 1 h. LF82, K12-C600 (orange), LF82 deletion mutants (green) and LF82 deletion mutants complemented by a plasmid expression the deleted gene (blue) were infected at a MOI of 30 which corresponds to 0–5 visible bacteria per macrophage at time point 1h (Fig 2A). Values represent the average of 3 to 7 experiments ± SD. Horizontal lines indicate viability decrease by 2, 5 and 10 fold compared to WT LF82. Data were analyzed using a Student’s *t* test: * *P* <0.05, ** *P* <0.01, *** *P* <0.001, **** *P* <0.0001 (black stars correspond to mutant vs WT, blue stars correspond to mutant vs its complementation).

We used THP1 (ATCC TIB-202) monocyte-derived into macrophages to monitor the population size of LF82 bacteria over a 24 h period post infection (P.I.) (Fig 1A). Colony-forming units (CFU) measurements revealed that after a long lag the LF82 population increased for 10–14 hours. Considering an exponential growth, the generation time would be τ = 0.15 h^-1^, 0.21 doubling / h. The population reached a maximum at 18–20 h of approximately 5-fold the value at 1 h; the number of LF82 then slightly decreased (reaching 2.5 -fold) at 24 h. In this environment LF82 might simultaneously encounter acidic pH [30], oxidative [31] and genotoxic stresses, toxic molecules such as antimicrobial peptides and a lack of important nutrients [32]. Surprisingly, the tolerance level of LF82 to any individual stress did not differ from a K12-C600 *E*. *coli* in *in vitro* conditions (S1 Fig). Using direct ex vivo Live and Dead labeling, it has been previously proposed that 80% of LF82 present at 24h within macrophages were alive [6]. We observed that this method slightly underestimates dead bacteria inside macrophages because of a weak propidium iodide (PI) labeling (S2A Fig). Live and dead assay performed immediately after macrophage lysis revealed a nearly constant proportion of dead LF82 in the population (20–30%) at 1 h, 12 h, 18h and 24 h post-infection (Fig 1A). THP1 Macrophages eliminate heat killed bacteria with a moderate decay rate of 0.6 h^-1^ (S2B Fig). This rate might be slightly underestimated because heat-killed LF82 differ from bacteria killed within phagolysosomes. We can therefore deduce that dead LF82 observed at 12 h, 18 h or 24 h did not correspond to the accumulation over infection period but rather to the bacteria killed in the last hours before observations. This finding led us to consider that LF82 must be under stress attack by macrophages at all times during infection, this permanent stress might explain the important CFU variability that we observed in different infections (Fig 1A and S2C Fig).

### LF82 is under attack by macrophages

Using RT-qPCR, we measured the expression of genes induced by the acid (*asr* and *ydeO*), oxidative (*soxS* and *ykgB*), and SOS (*sulA*) responses, and the responses to membrane alteration (*pspB*), the lack of Mg^2+^ (*mgrB*), the lack of phosphate (*phoB*) and the *gltT* tRNA gene that is repressed by the stringent response (Fig 1B). Every response pathway was significantly induced inside the macrophage. The induction of acid and oxidative responses, the response to membrane alterations and to the lack of Mg^2+^ was already high at 1 h P.I., while the SOS response, was only manifest at 6 h and 24 h P.I.. The expression of the *gltT* tRNA was repressed at 1 h P.I. indicating that stringent response is on early in the infection and dissipates at later time points.

### Environmental stresses influence LF82 survival

To test the impact of stress responses on the ability of LF82 to colonize macrophage, we constructed deletion mutants of several key regulators of *E*. *coli* stress pathways and analyzed their survival. Deletion of the acid stress regulators *evgA-evgS*, *phoP* and *ydeO* significantly impacted the ability of LF82 to survive and multiply within macrophages to a level comparable to or even below that of a K12-C600 *E*. *coli* (Fig 1C). Similar observations were obtained with the *rpoS* (general stress response), *recA* (SOS response), *soxS* (oxidative stress) and *pspA* and *htrA* (envelope damages) deletion mutants. The ppGpp0 strain, *relA spoT* deletions, impaired in the stringent response reporting a lack of nutrients, is the most impacted strain; less than 5% of the initial population survived a 24 h period within macrophages. Survival of the *ydeO*, *relA spoT*, *dksA* and *recA* deletion strains was respectively rescued by the expression *ydeO*, *spoT*, *dksA* and *recA* from a plasmid suggesting that these genes are directly involved in survival. Interestingly, even if the viability of the *recA* mutant was only reduced by two fold compared to the WT strain, we observed that the majority of colonies formed on LB plates 18h after macrophage lysis were 2–4 times smaller than those formed by the WT strain and the *recA* mutant itself without stress. It suggests that RecA and maybe DNA repair are important for the recovery of a normal growth rate following macrophage attacks. These observations confirmed that LF82 encounter severe stresses in the macrophage environment and that its ability to repair stress mediated injuries will determine survival.

### SOS and stringent responses severely impacted LF82 survival

Because of their known potential to influence growth and cell cycle parameters we explored the stringent and SOS responses in more details. RecA is the main inducer of the SOS response, which activates nearly 100 genes involved in DNA repair and many others with unrelated or unknown functions, but it is also crucial to correct DNA lesions by homologous recombination and translesion synthesis [33]. In addition to *recA* deletion, we constructed deletions of *sulA* (division inhibitor) and a mutation in *lexA* (*lexAind-)*, which blocks SOS induction in MG1655 *E*. *coli* and reduces viability in the presence of mitomycin C for LF82 and MG1655 (S3A Fig). We observed that the deletion of each SOS gene significantly decreased the survival of LF82 within macrophages (Fig 1C). Inside the macrophage, survival of the ppGpp0 strain was dramatically impacted. However, this mutant also presented a strong growth defect in liquid culture that complicates interpretation of the macrophage results. To study the impact of the stringent response on LF82 survival and induced antibiotic tolerance, we constructed deletion mutants that might have partial stringent response phenotypes; deletion of *dksA*, encoding a protein linking the stringent response to transcription [34]; and deletion of the polyphosphate kinase and exopolyphosphatase *ppk* and *ppx* [35]. As expected, the *dksA and ppk-ppx* deletions had a much less dramatic effect on LF82 growth and survival within macrophages than the *relA-spoT* mutant; nevertheless, the *dksA* mutation significantly impacted the number of live bacteria recovered at 24 h P.I. (Fig 1C). We investigated the ability of LF82 to survive within macrophages when both stringent and SOS responses were altered. We chose to combine *dksA* deletion with *recA* deletion or *lexAind-* mutation. These strains presented a survival defect comparable to that of the single *dksA* mutant (Fig 1C). These observations demonstrate that surviving LF82 simultaneously or successively require SOS and stringent responses.

### Inside phagolysosomes individual LF82 were not homogenously responding to stresses

**Fig 2:**
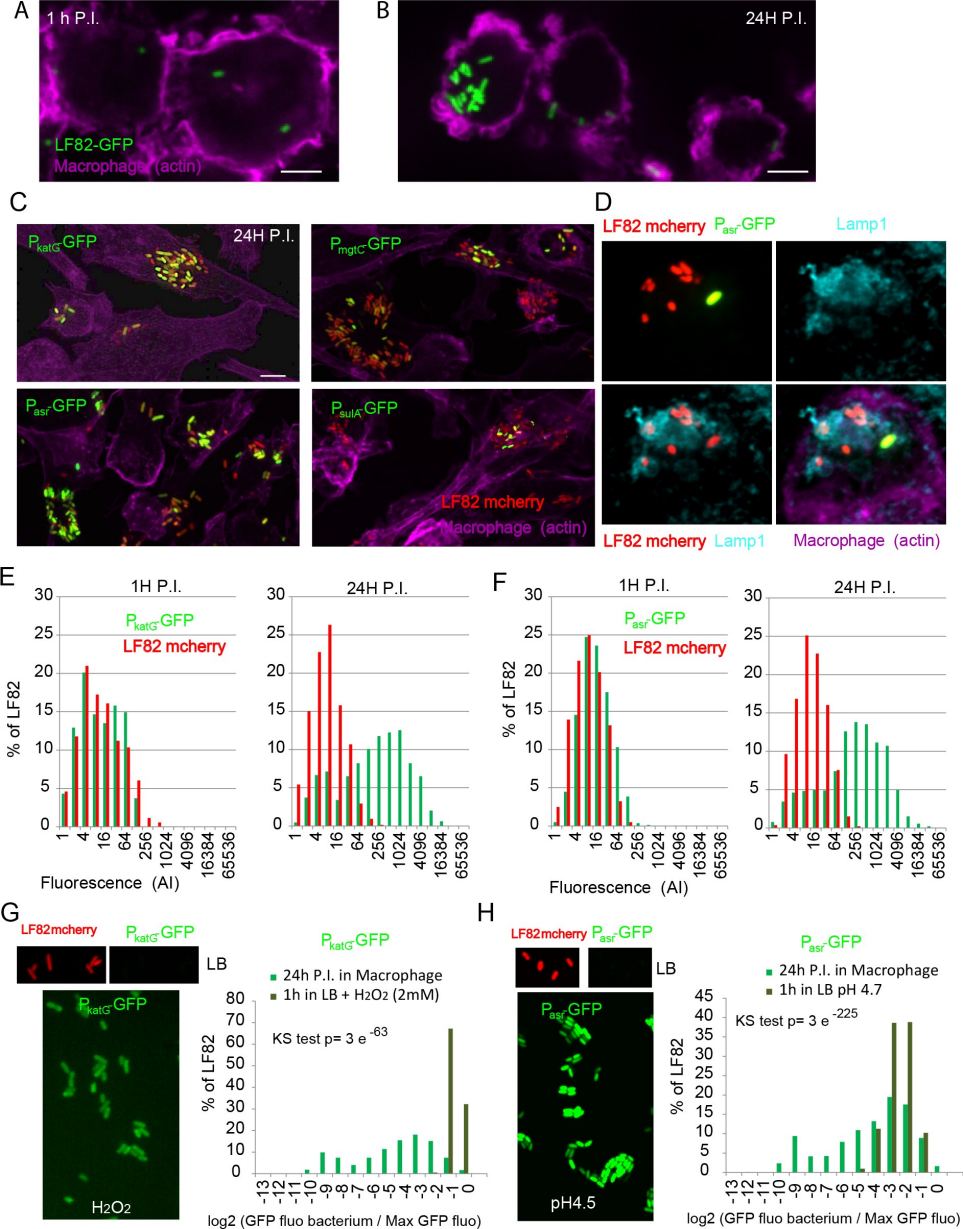
Intracellular LF82 show heterogeneous stress responses. A) Imaging of THP1 macrophages infection by LF82-GFP at a MOI of 30. Representative images at 1 h P.I.. Scale bar is 5 μm. B) Imaging of LF82-GFP at 24 h P.I.. C) Imaging of LF82-mCherry stress responses at the single cell level with biosensors. Imaging was performed at 24 h P.I.. LF82-mCherry was transformed with plasmids containing either the *katG* promoter fused to GFP (P*katG-*GFP), the *mgtC* promoter (P*mgt*C-GFP), the *asr* promoter (P*asr-*GFP) or the *sulA* promoter (P*sulA-*GFP). D) Imaging of LF82-mCherry P*asr*GFP and Lamp1 phagolysosome marker. E) Measure of the fluorescence intensity of individual LF82-mCherry containing the *katG* promoter fused to GFP at 1 h and 24 h P.I. Fluorescence was measured on images acquired on fixed bacteria immediately after macrophage lysis (N = 2000) with a spinning disk microscope F) Measure of the fluorescence intensity of individual LF82-mCherry containing the *asr* promoter fused to GFP at 1 h and 24 h P.I. (N = 2000). G) LF82 P*sulA*-GFP, LF82 P*katG*GFP, LF82 P*asr-*GFP in LB and 1h after addition of MMC (5μM), H2O2 (5μM) or switched to pH4.7 1h before imaging. Distribution of the fluorescence of LF82 P*asr-*GFP after 24h post infection in macrophage (from panel B) and after 1 hours of growth in LB buffered at pH4.7. Fluorescence values were expressed as their log2ratio with the average value of the maximum decile (maximum expression). Distributions were compared with a Two-sample Kolmogorov-Smirnov (KS) test.

Imaging revealed great heterogeneity in the number of LF82 bacteria within individual macrophages. At 18 h or 24 h P.I., many macrophages presented fewer than 5 bacteria, which was comparable to the amount observed at 1 h P.I. (Fig 2A); however, a number of macrophages also presented foci containing up to 50 bacteria (Fig 2B). These observations led us to consider that LF82 were not homogeneously stressed by macrophages. We used GFP fusion with selected stress response promoters to monitor variability of these responses among bacteria and among macrophages (Fig 2B). For acidic, oxidative, SOS and the lack of Mg^2+^ responses we observed GFP positive and GFP negative bacteria at 24 h P.I.. As illustrated on Fig 2D, we observed phenotypical heterogeneity even in a single Lamp 1 positive vacuolar environment. We measured the GFP fluorescence as a proxy of stress response induction inside macrophages for the P*asr* and P*katG* promoters (Fig 2E and 2F). For both reporters we observed that GFP fluorescence distributed along a 200 fold range at 24 h P.I.. The heterogeneity in GFP fluorescence for intracellular bacteria was far larger than that observed for cultures in acidic LB (16 fold) or LB with H_2_O_2_ (4 fold)(Fig 2G and 2H). At 24 h P.I., we found that approximately 30% of the bacteria had poorly responded to oxidative or acid responses (Fig 2E and 2F). Owing the high stability of the GFP protein that we used in this assay, it is unlikely that this heterogeneity resulted from short pulses of induction separated by long repression periods. The bacteria showing the weaker stress response might actually be dead. To test for this we monitored the ability of LF82 *Pasr-GFP* and LF82 *PkatG-GFP* bacteria to form colonies on LB agarose pad after macrophage lysis (S4 Fig). Although we frequently observed that bacteria with the weakest GFP signal were not able to form colonies, we also observed GFP positive bacteria that failed to divide and bacteria with a weak GFP that restarted growth rapidly. We observed respectively for P*asr-GFP* and P*katG-GFP* 30 +/- 15% and 26+/- 17% of bacteria that are not able to divide within 2.5 h of recovery time. This number is in good agreement with the live and dead assay (Fig 1A). Interestingly this experiment revealed that the lag time before the first division varies from 20 min to 140 min suggesting that LF82 has to repair lesions before returning to growth. We therefore questioned whether heterogeneity in stress response might reflect the coexistence of bacteria in different physiological states and perhaps cell growth or cell cycle regulations.

### Macrophages induce the formation of non-growing LF82

**Fig 3:**
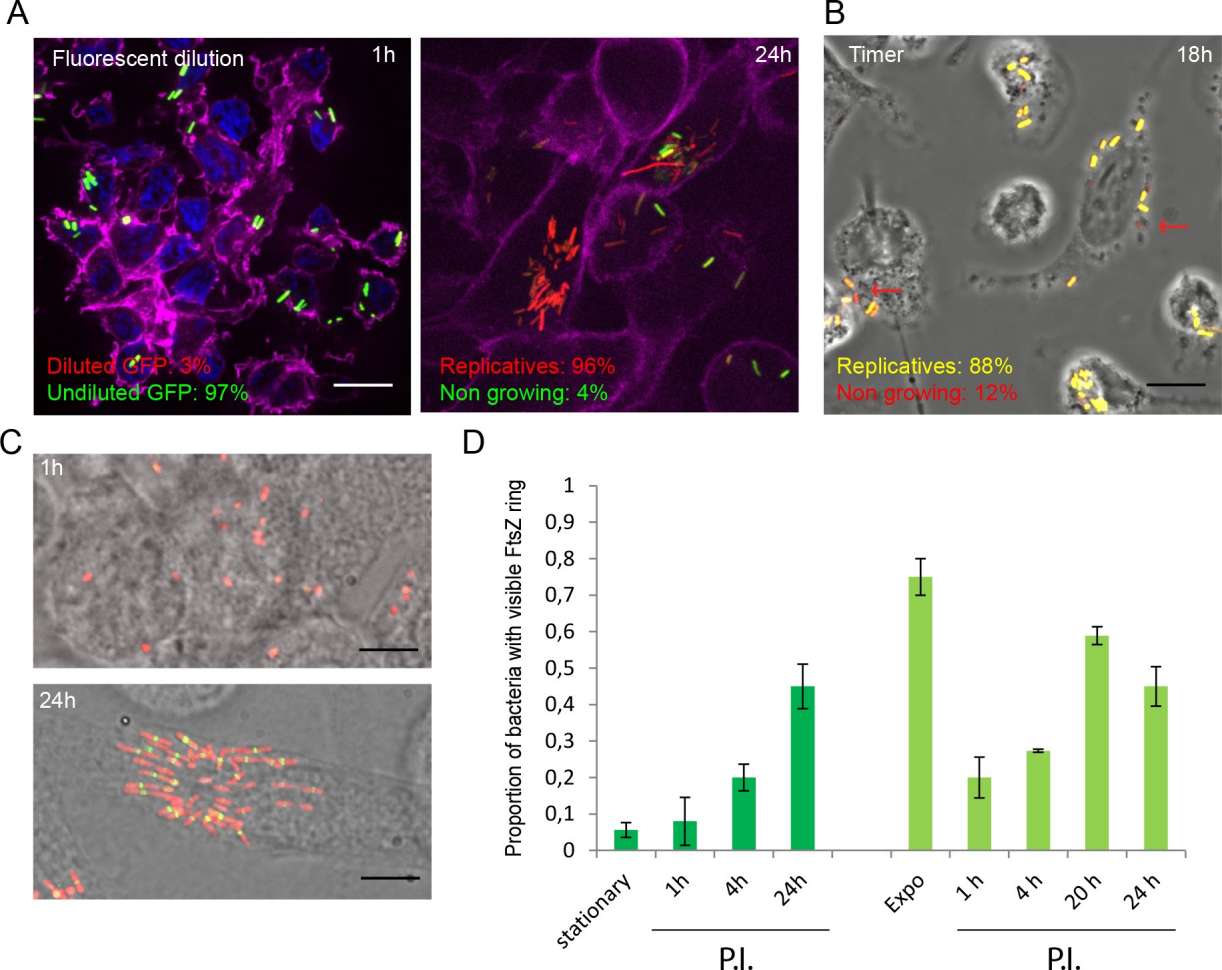
Non-growers LF82 are produced during intracellular growth. A) Representative image of LF82 containing the fluorescent dilution plasmid (pFC6Gi) at 1 h and 24 h post-infection. The frequency of replicative and non-growing LF82 (undiluted GFP) is indicated (N = 300). B) Representative image of LF82 containing the TIMER plasmid (pSC101-TIMER) at 18 h post-infection. The red arrows points toward the reddest LF82. The frequency of replicative and non-growing LF82 is indicated (N = 300). C) Representative images of LF82-mCherry FtsZ-GFP at 1 h and 24 h post-infection. Infections presented in panels A to C were performed with a stationary phase culture of LF82 (O.D. 2). Scale bars are 5 μm. D) Measure of the frequency of LF82 presenting a FtsZ ring in populations growing in LB or within macrophages (N = 300).

We analyzed heterogeneity in LF82 cell cycle within macrophages by using two complementary fluorescence assays. First, Fluorescent Dilution (FD) highlights bacteria that have not divided since the time of infection (Fig 3A and S5A–S5C Fig). FD consists in a plasmid expressing mCherry under a constitutive promoter and GFP under an arabinose inducible promoter. When induction is stopped at the moment of infection the production is stopped and replicating bacteria will dilute GFP at each division; in non-growing bacteria the stable GFP fluorescence is kept for several days. FD provides a likelihood of the cells going through a certain number of divisions. The maximum dilution detectable by the assay in our conditions is 8 generations in LB (S5A and S5B Fig). With this calibration we estimated that a subset of the LF82 population underwent around 5–6 divisions at 24 h P.I.. In good agreement with the CFU, measurement of FD showed that only 20% of the population has performed more than 1 division at 6 h P.I. From these observations, we can estimate that the highest generation rate of LF82 within macrophages is ≈ 0.5 doubling /h between 6 and 20 h P.I.. Interestingly, FD also revealed that approximately 4% of the population did not divide or divided fewer than 2 times intracellularly in 24 h (Fig 3A and S5B Fig). By contrast among the small amount of K12-C600 bacteria that survived for 24 h in the macrophage, 60% of K12-C600 bacteria underwent fewer than 2 divisions and less than 10% underwent 5 divisions (S5D Fig). Second, we used TIMER to refine these observations. TIMER is based on the maturation of a mutant version of dsRED, the slow growing bacteria accumulate red fluorescence, and therefore it provides an instantaneous evaluation of the generation time during the infection kinetics (Fig 3B and S6 Fig). TIMER indicated that at 18 h P.I., 10% of the LF82 population was not actively dividing (S6 Fig), supporting the existence of a non-growing or slow-growing subpopulation. Since they require dilution of fluorescent proteins both TIMER and FD are not informative about the first hours of the infection. Therefore, we used a GFP fusion with the septal ring protein FtsZ to monitor division in the individual bacterium (Fig 3C). In LB, exponentially growing LF82 frequently presented the FtsZ ring (70% of the population), but stationary phase LF82 rarely presented the FtsZ ring (<2% of the population, Fig 3D). Following infection of macrophages with stationary phase culture of LF82 *ftsZ-gfp*, we observed that 5% (+/-2) and 40% (+/- 12) of the population, respectively, presented an FtsZ ring at 1 h and 24 h P.I. (Fig 3C and 3D). Following infection with exponentially growing LF82 we observed a sudden reduction in the number of LF82 presenting an FtsZ ring at 1 h, this number is maintained at a low level 4h P.I. (Fig 3D). The three reporters (FD, TIMER and FtsZ) provided complementary indications: i) within macrophages, LF82 strongly slow down their cell cycle for several hours; ii) starting at 6 h P.I. LF82 multiply; in this phase the generation time may vary among bacteria but can be as short as 2h; iii) a part of the population, completely halts their cell cycle and become non-grower. The difference between the number of non-growing LF82 revealed by TIMER and FD shows that non-growing LF82 are formed late in the infection kinetics and not only upon phagocytosis.

### Macrophages induce the formation of antibiotic-tolerant LF82

**Fig 4:**
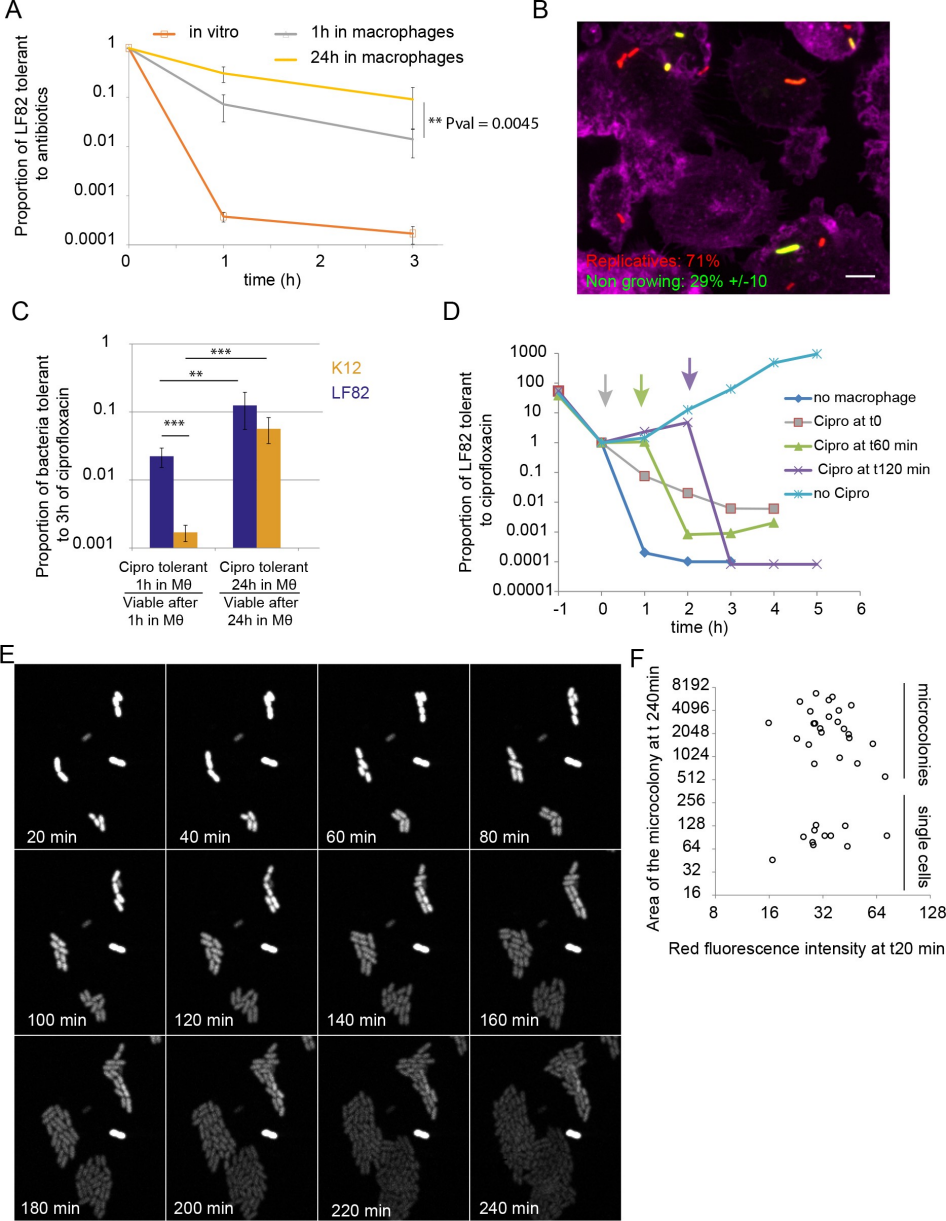
Persisters LF82 are produced during intracellular growth. A) Measure of the proportion of LF82 that were tolerant to ciprofloxacin (10x MIC) at 1 h or 3 h. LF82 were cultivated up to OD 0.3 in LB medium (*in vitro*) or harvested after 1 h or 24 h post infection within macrophages. The challenges exerted on bacteria having spent 1h or 24h through macrophages started immediately after macrophage lysis (see experimental procedures). Data were analyzed using a Student’s *t* test to determine differences with the proportion of ciprofloxacin-tolerant LF82 after a 24 h passage compared to a 1 h passage, **P < 0.01. B) Proportion of non-growing LF82 (labeled using the Fluorescent Dilution assay) observed within macrophages (24 h P.I.) following a 6 h ofloxacin (10μg/ml) treatment. C) Ratio of ciprofloxacin-tolerant versus viable LF82 and K12-C600 bacteria after macrophage infection. Values are averages of 5 experiments. Data were analyzed using a Student’s *t* test to determine differences with the proportion of ciprofloxacin-tolerant LF82 at 1 h post-infection, *P < 0.05 and ** *P*<0.001. D) Measure of the proportion of LF82 that were tolerant to ciprofloxacin at 1 h or 3 h with increasing times after macrophage lysis before ciprofloxacin addition. The data presented on this graph are representative of two independent experiments E) Imaging of the regrowth properties of individual LF82-TIMER bacteria after macrophages lysis. Infections were performed for 20 h and then macrophages were lysed, LF82 spread on to LB agarose pads and immediately imaged at 37°C. TIMER red fluorescence was progressively lost as microcolonies formed. The lysis procedure requires 20 minutes before the first field can be observed (t20 min). F) Measure of the ability of LF82 to form microcolonies as a function of red TIMER fluorescence at t20 min of the experiment are presented in E; areas of microcolonies are expressed in pixels^2^.

Non-growing Salmonella phenotypes have been observed inside macrophages and during mouse infection [16,36]. Being tolerant to subsequent antibiotic challenge they were recognized as persisters. We inquired whether also for AIEC LF82 the non-growing component of the population had enhanced antibiotic tolerance. In exponentially growing liquid cultures, approximately 0.01% LF82 tolerated a 3 h ciprofloxacin challenge, and can therefore be considered persisters (Fig 4A). Following a brief passage through the macrophage, the frequency of LF82 bacteria tolerant to ciprofloxacin increased to 0.5% (nearly 50 fold compared to exponentially growing LF82). Interestingly the number of tolerant LF82 reached 5% (500 fold compared to exponentially growing LF82) after 24 h in the macrophage (Fig 4A). This near to 10-fold increase in the number of tolerant bacteria after a 24 h intracellular infection compared to a 1 h infection indicates that, like non-growers, tolerant phenotypes are not exclusively formed upon infection, but also as bacteria multiply inside the macrophage. In this respect, the behavior of LF82 differs significantly from *S*. *typhimurium*, which forms a large number of persisters upon macrophage entry, but this population remains stable during the infection [16]. To test if non-growing LF82 revealed by the FD assay indeed corresponded to the antibiotic-tolerant population, we used the macrophage-permeable antibiotic ofloxacin [37]. We added ofloxacin (10x MIC) for 4 h after 20 h of intracellular growth, and we observed significant increases in the proportion of green LF82 (non-growing) inside macrophages (Fig 4B). These observations suggest that the sub-population of non-growing bacteria largely overlaps with that of persisters, where protection from intracellular stresses may also confer enhanced tolerance to antibiotics.

### Tolerance to antibiotics is enhanced for LF82 compared with K12-C600 *E*. *coli*

We compared the tolerance to ciprofloxacin for LF82 and a non-pathogenic K12-C600 laboratory strain following macrophages passages. After a brief passage in macrophages, the proportion of LF82 that were tolerant to ciprofloxacin was significantly higher for LF82 than K12-C600 (Fig 4C). Even if the absolute number of ciprofloxacin-tolerant K12-C600 was largely reduced compared with LF82, their proportions among bacteria that survived 24 h inside macrophages were comparable (Fig 4C). These findings demonstrate that the number of antibiotic-tolerant bacteria formed in response to macrophage attack is reinforced for LF82 compared with the laboratory strain.

### Tolerance to antibiotics is a transient state

We next evaluated whether the antibiotic tolerance was a stable or transient phenotype. We used the macrophage lysis procedure to recover LF82 with induced persistence for 1 h in the macrophage; then, we either challenged them immediately with ciprofloxacin or allowed them to recover in LB for 1 h or 2 h before antibiotic challenge. When bacteria were cultured for 1 h in LB, the frequency of tolerant bacteria was decreased in comparison to bacteria that were immediately treated with the antibiotic; however, this number was still higher than that of bacteria that had not infected macrophages. Two hours in LB was sufficient to cause a comparable frequency of ciprofloxacin-tolerant LF82 to that of bacteria that had not encountered macrophages (Fig 4D). These observations show that when the environment is no longer stressful, antibiotic-tolerant, non-growing LF82 rapidly switch back to a replicative mode.

### Characterization of non-growing LF82

To evaluate the proportion of antibiotic persisters in the non-growing population observed with the fluorescence assays, we infected macrophages with TIMER-tagged LF82, lysed the macrophages and allowed bacterial growth on a LB-agarose pad under the microscope at 37°C. Seventy percent of the LF82 bacteria recovered quickly from the challenge and formed microcolonies, but approximately 30% of them never divided (Fig 4E). Some of these non-cultivable LF82 presented non-growing TIMER fluorescence (Fig 4F). The non-growing population is therefore not homogeneous and only a portion of it can contribute to persister formation. A similar observation was described for S. typhimurium labeled with FD [16].

### SOS and stringent responses influence antibiotic tolerance

**Fig 5:**
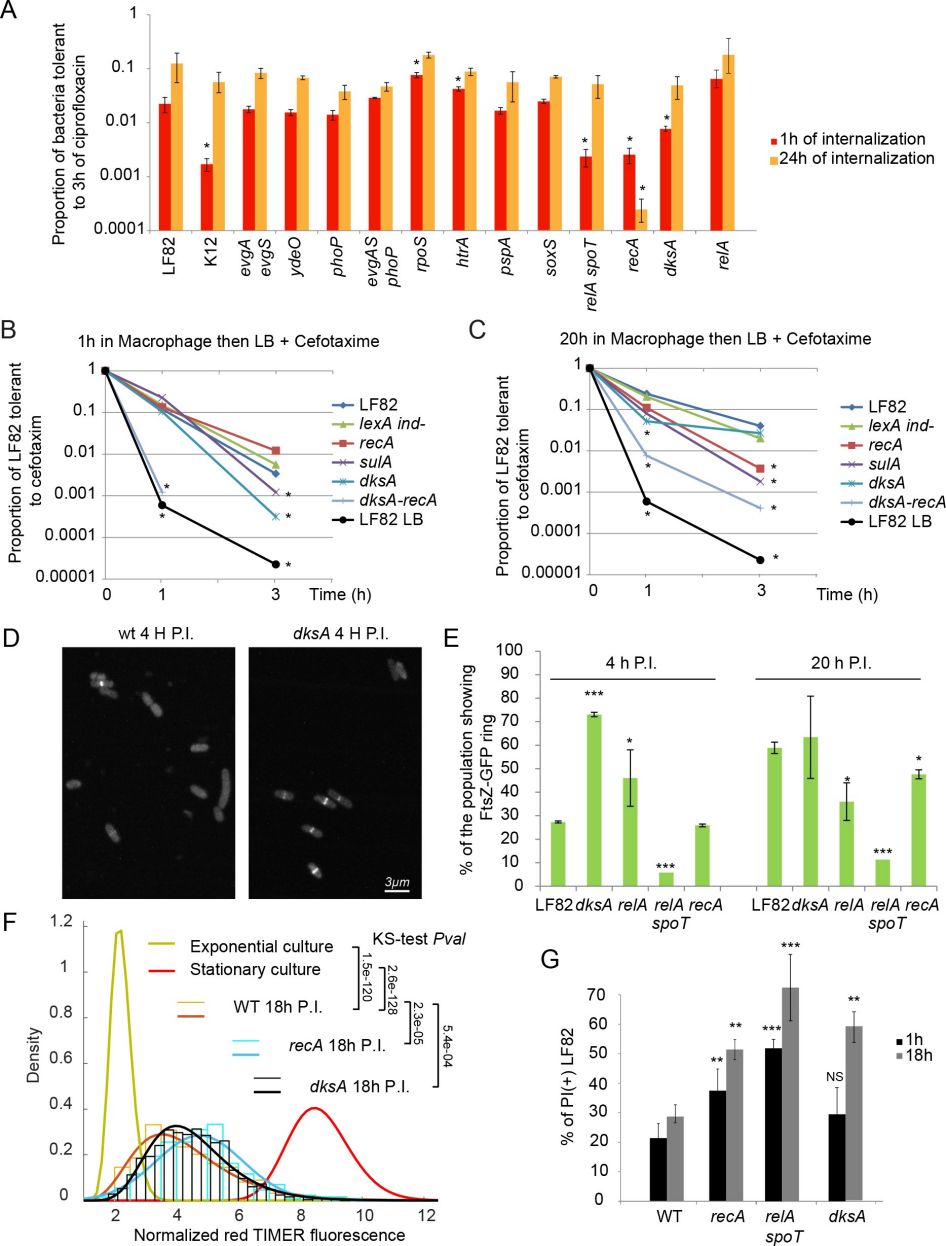
SOS and stringent responses control LF82 cell cycle in the macrophages. A) Proportions of LF82, K12-C600 and LF82 deletion mutants that were tolerant to a 3h ciprofloxacin challenge following a 1 h or 24 h intracellular period within THP1 macrophages. Values represent the average of 3 to 7 experiments. Data were analyzed by using a Student’s *t* test to determine differences compared with wild-type LF82. *P < 0.05. B) Measure of the proportions of LF82 *lexA ind*, *recA*, *sulA*, *dksA*, *dksA lexAind-*, *dksA recA* mutants that were tolerant to 3 h of cefotaxim challenge after 1 h of macrophage infection. The 3 htime point was below detection limit for the *dksA recA* mutant because of the poor viability of the mutant in macrophages. Cefotaxime only kills growing bacteria; therefore we resuspended LF82 in LB after macrophage lysis. Under these conditions, we did not observe the plateau observed for persisters to ciprofloxacin, suggesting that tolerant rather than persister bacteria were measured. C) Same as in B but with 20 h of macrophage infection. Values represent the average of 3 to 7 experiments. Data were analyzed using a Student’s *t* test to determine differences compared with wild-type LF82. *P < 0.05. D) Imaging of FtsZ-GFP ring for the WT and *dksA* strains after macrophage lysis 4 h P.I. E) Measure of the number of dividing bacteria (presence of an FtsZ-GFP ring at mid-cell) for the WT, *dksA*, *relA*, *relA-spoT* and *recA* strains after macrophage lysis at 4 h and 20h P.I. N = 3, 300 bacteria were counted for each experiment. Data were analyzed using a Student’s *t* test to determine differences with the proportion LF82 cells with a FtsZ ring at each time point, *P < 0.05 and *** *P*<0.0001. F) Distribution of red TIMER fluorescence in exponential and stationary phase culture and 18 h P.I.. The WT and *recA* and *dksA* mutants were tested during infection. Data are fitted with normal law (thick lines). Data were analyzed two by two with a Kolmogorov-Smirnov tests. G) Live and dead assay performed 1 h and 18 h P.I., in the LF82, LF82*recA*, LF82*lexAind-*, LF82*sulA*, LF82*relAspoT* and LF82*dksA* strains. Data were analyzed using a Student’s *t* test to determine differences with the proportion of Propidium iodide (+) LF82 at 1 h and 18h post-infection, ** *P*<0.001 and *** *P*<0.0001.

Among mutants that affected LF82 survival (Fig 1C), only the *recA*, *relA spoT* and *dksA* deletions negatively impacted the number of LF82 that were tolerant to a 3h ciprofloxacin treatment (Fig 5A). The impact of the *recA* deletion might be misinterpreted because ciprofloxacin alters DNA and limits the ability of *recA* persisters to form colonies on LB plates. Therefore, we repeated the tolerance assay with cefotaxime for the following SOS mutants: *recA* (impaired for DNA lesion repair and SOS induction), *lexAind-* (unable to induce SOS) and *sulA* (unable to block cell division). I*n vitro*, SOS mutants did not present defect for cefotaxime tolerance (S3B Fig). However, these mutants exhibited a decreased tolerance to antibiotics when persisters were induced by a pretreatment with subinhibitory concentrations of ciprofloxacin (S3C Fig). This finding is in good agreement with previous reports [19], and it confirms that SOS induction favors the production of persisters. We analyzed cefotaxime tolerance of these SOS mutants after a 1 h or 20 h period within macrophages (Fig 5B and 5C). We observed a significant reduction of the proportion of *recA* and *sulA* mutants that were tolerant to cefotaxime treatment after a 20 h passage in the macrophage (Fig 5C) but no effect on bacteria that remained only 1 h in macrophages (Fig 5B). The *lexAind-* mutation did not change the number of tolerant bacteria in these conditions. We also analyzed the *dksA* mutant in these assays; surprisingly, it behaved differently than the *recA* and *sulA* mutants: we observed a significant reduction of the proportion of cefotaxime tolerant bacteria following a 1 h passage within macrophages (Fig 5B) but not when the bacteria remained for 20 h in macrophages (Fig 5C). To test an eventual epistatic relation between SOS and stringent response we combined *recA* and *dksA* deletions. The deletion of *recA* exacerbated the impact of *dksA* deletion on the ability of LF82 to become tolerant to cefotaxime after a brief infection (Fig 5B). The deletion of *dksA* exacerbated the impact of *recA* deletion at 20 h P.I. (Fig 5C). Our observations suggest that production of persister/tolerant LF82 bacteria is mainly under the control of the stringent response in the first hours of infection but mainly controlled by genotoxic stress, the SOS response and DNA lesion processing later in the infection. When one of these two responses is deficient the rare production of persister /tolerant LF82 requires the other.

### The role of SOS response and stringent response for the control of LF82 cell cycle in the macrophages

Knowing that SOS and stringent responses influence the production of antibiotic tolerant LF82 after a passage within macrophage we examined whether they also contributed to LF82 growth control, i.e. production of dividing, non-growing, replicative or dead LF82. We used FtsZ-GFP fusion to measure the number of dividing cells during infection in the *dksA*, *relA*, *relA-spoT* and *recA* mutants. We observed an increased number of cells presenting an FtsZ ring in the *dksA* and *relA* mutants 4 h P.I. (Fig 5D and 5E). This suggests that when stringent response is altered LF82 cannot immediately curb its cell cycle upon phagocytosis. The number of bacteria with an FtsZ ring was really low in the *relA spoT* mutant, we believed this was a consequence of its poor viability (Fig 5E). The Live and dead assay confirmed that the majority of the population is dead when stringent response is completely abolished (Fig 5G). The number of dividing bacteria 4 h P.I. was unchanged for the *recA* mutant (Fig 5E). This is in agreement with the absence of effect of the *recA* deletion on the production of cefotaxime tolerant LF82 early in the infections. At time point 20 h P.I. the percentage of dividing bacteria increased strongly for the WT, increased to a lesser extent for the *recA* mutant and remained constant for the *dksA* and *relA* mutants. Next we evaluated growth rate in the *recA* and *dksA* mutants with the TIMER tool. The distribution of red TIMER fluorescence was significantly changed for the *recA* and *dksA* mutants compared to the WT strain (Fig 5F). The *recA* deletion shifted the distribution of the population toward red fluorescence (the mean red fluorescence of the *recA* population is 4.7 compared to 4.2 for the WT, KS-test P = 2.3x10^-5^) suggesting that the average growth rate of the population is reduced. This observation is in good agreement with the reduced number of *recA* bacteria presenting FtsZ ring (Fig 5E). In the *dksA* mutant, the distribution was also slightly shifted toward higher amounts of red fluorescence (mean = 4.5, KS-test P = 5.4x10^-4^) compared to the WT (Fig 5E). This suggests that in the absence of DksA the average growth rate is reduced, however in this case this is not correlated with a reduction of the number of bacteria presenting an FtsZ ring (Fig 5E). The proportion of bacteria replicating rapidly is reduced both in the *recA* (11%) and *dksA* (13%) mutants compared to the WT strain (27%) (S6C Fig). The number of bacteria with the highest red TIMER fluorescence, the fluorescence that should correspond to non-growers, is marginally changed in the two mutants compared to the WT strain (S6C Fig). Finally, the live and dead assay showed an increased lethality of the *recA*, *relA spoT* and *dksA* mutants at both time points (Fig 5G) suggesting that, in the macrophage environment, failing growth and division controls will frequently lead to LF82 death.

Altogether our results showed that the stringent response is the main controller of the early intracellular survival of LF82; it limits LF82 cell division in the early phase of the infection, putatively induces the formation of non-growers and among them persisters. Later on, when replication is resumed, SOS response grows in importance. DNA lesions that have been accumulated in the lag phase must be repaired to allow replication and formation of new non-growers and new persisters.

### Kinetics of macrophage infection

**Fig 6:**
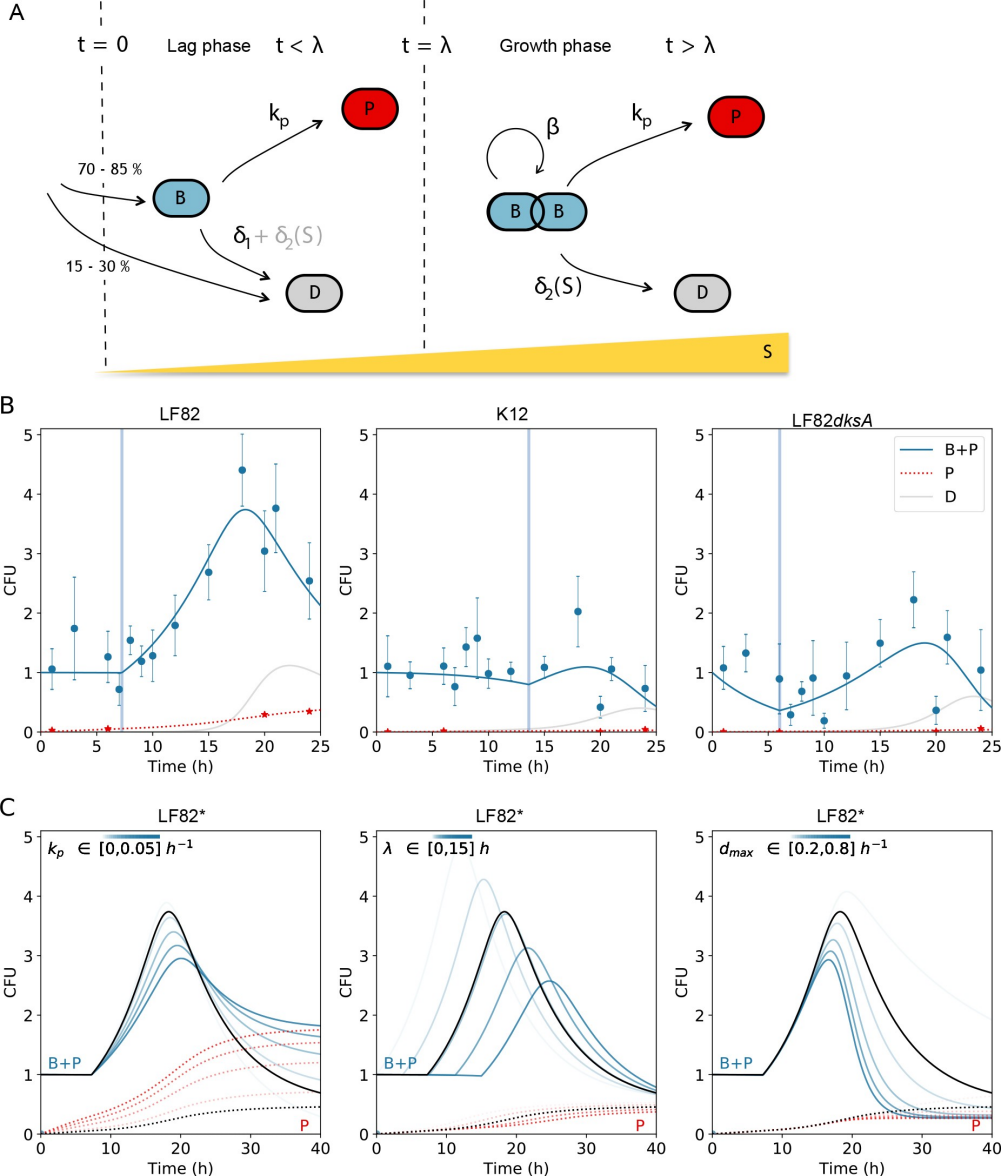
Kinetics of macrophage infection. A) Model of infection of THP1 macrophages by LF82, describing the processes of net growth, switch to persistence and stress-induced death as explained in the text. Part of the cells that enter the macrophage die at the onset of the infection (t = 0). During lag phase (0<t<λ), death either exactly compensates birth or, in the mutant lacking the stringent response, results in a negative net growth rate -δ_1_. Later in the infection, the net growth rate β is positive. Death rate due to stress accumulation (yellow bar) is negligible in the early stages of infection and becomes particularly important at late time points. Bacteria switch to a persistent state at a rate k_p_ independent of the growth stage. B) Experimental measures of the infection kinetics (CFUs, circles; persister fractions, stars) collected over 24h for LF82 (this set of data is already presented on the Fig 1A), *E*. *coli* K12-C600 and LF82 *dksA* (data obtained on one set of THP1 macrophages in triplicates) and the best fitting parameters (S1 Table) of model eq. (1) (Methods). LF82 data corresponds to the data presented on Fig 1A, *E*. *coli* K12-C600 and LF82 *dksA* data are median of 3 to 5 infections per time point. Continuous lines represent total number of bacteria (B+P, continuous line) and persisters (P, dotted line). Vertical lines indicate the duration λ of lag phase. C) Projected changes in infection dynamics for ‘virtual mutants’ LF82*, obtained by varying k_p_, λ and d_max_−the parameters that quantitatively differ between LF82 and *E*. *coli* K12-C600 –around the LF82 best fit solution (black line); colored lines correspond to parameter values within the indicated interval.

AIEC LF82 tolerates macrophage induced stresses, thus it survives and multiplies in the phagolysosome. The population expansion is accompanied by a rise in the number of bacteria that do not grow and tolerate an antibiotic challenge (called henceforth persisters). The change in time, during macrophage infection, of LF82 population size and fraction of persisters are relevant to future fundamental studies, but also to devising therapeutic strategies involving AIEC or other intracellular pathogens. In order to explore the mechanistic bases of the infection kinetics, we have used a mathematical model (Fig 6A) to fit the observed changes in CFU and persister counts during 24 h for LF82, K12-C600 and the stringent response mutant LF82*dksA* (Fig 6B). The model is based on the following biologically-informed hypotheses (illustrated in Fig 6A and detailed in the Supplementary text 1). i) Reproduction: the population of replicating bacteria B has a constant net growth rate (birth minus death rate) δ_1_, which is either 0 or negative during a lag phase of duration λ, and β>0 otherwise. ii) A stress-induced death rate, δ_2_(S) that increases with stress (S). We assume that stress S, possibly due to lesions (DNA lesions, membrane alterations, oxidative damages among others) that accumulate over time or due to a macrophage response to the infection, builds up in time proportionally to the total number of bacteria in the population. iii) Switch to persistence: bacteria have a constant rate k_p_ of generating non-growing, stress-tolerant phenotypes P. The dynamics of a population of bacteria can be described by a set of three ordinary differential equations for the number B of non-persister bacteria, the number P of persisters, and the stress variable S (Methods). The number of dead bacteria D can be derived from these under the assumption that dead LF82 bacteria decay exponentially with rate 0.56 (computed from the assay in S2C Fig). The model has a total of 12 parameters for the three strains, which are fitted to data as explained in Methods and S1 Text. Although LF82 displays a considerable overshoot in population size (as also observed in [2]), the dynamics can be reproduced by choosing the same β for every strain. We estimated such net growth rate to 0.15 ± 0.003 h^-1^, corresponding to 0.21 divisions per hour (Fig 6B), consistent with independent cell-level measures by FD (S5 Fig). The most notable quantitative difference between strains is that K12-C600 displayed a lag phase of more than 13 h, twice as long as LF82 and LF82*dksA*. A consequence of this difference is that when K12-C600 bacteria start actively duplicating, stress has already built up. Together with K12-C600’s enhanced sensitivity to stress, this curbs the population expansion, resulting in a lower overall growth within macrophages. In the LF82*dksA* mutant, growth is instead impaired by increased initial mortality (whose rate δ_1_ has been estimated by PI measures, S2B Fig), presumably related to stringent response failure. With respect to the other strains, moreover, LF82 is advantaged at later times–when the SOS response becomes important–thanks to reduced stress-induced death rate. Rate of persistence production for LF82 and LF82 *dksA* (0.08 and 0.002 h^-1^, respectively) is estimated to be higher than for K12-C600 (0.001 h^-1^), supporting the notion that AIEC strains within macrophages turn to persisters at an enhanced rate, but less so if their stringent response is impaired. The model allows testing changes in infection dynamics for ‘virtual mutants’ LF82*, obtained by varying k_p_, λ and d_max_−the parameters that quantitatively differ between LF82 and *E*. *coli* K12-C600 (Fig 6C). The total population overshoot is enhanced when lag phase is shorter and the effect of stress less acute, but damped when persisters production is more frequent. Interestingly, the phenotypes of the single stress response mutants (acid, oxidative, lack of Mg^2+^), i.e. reduced CFU at 24 h without perturbation of the persister proportion in the population (Figs 1C and 3A) were nicely reproduced by a change in the single d_max_ parameter. This suggests that the model can be used to plan future works on the effect of mutants or drugs on macrophage colonization by AIEC.

## Discussion

**Fig 7:**
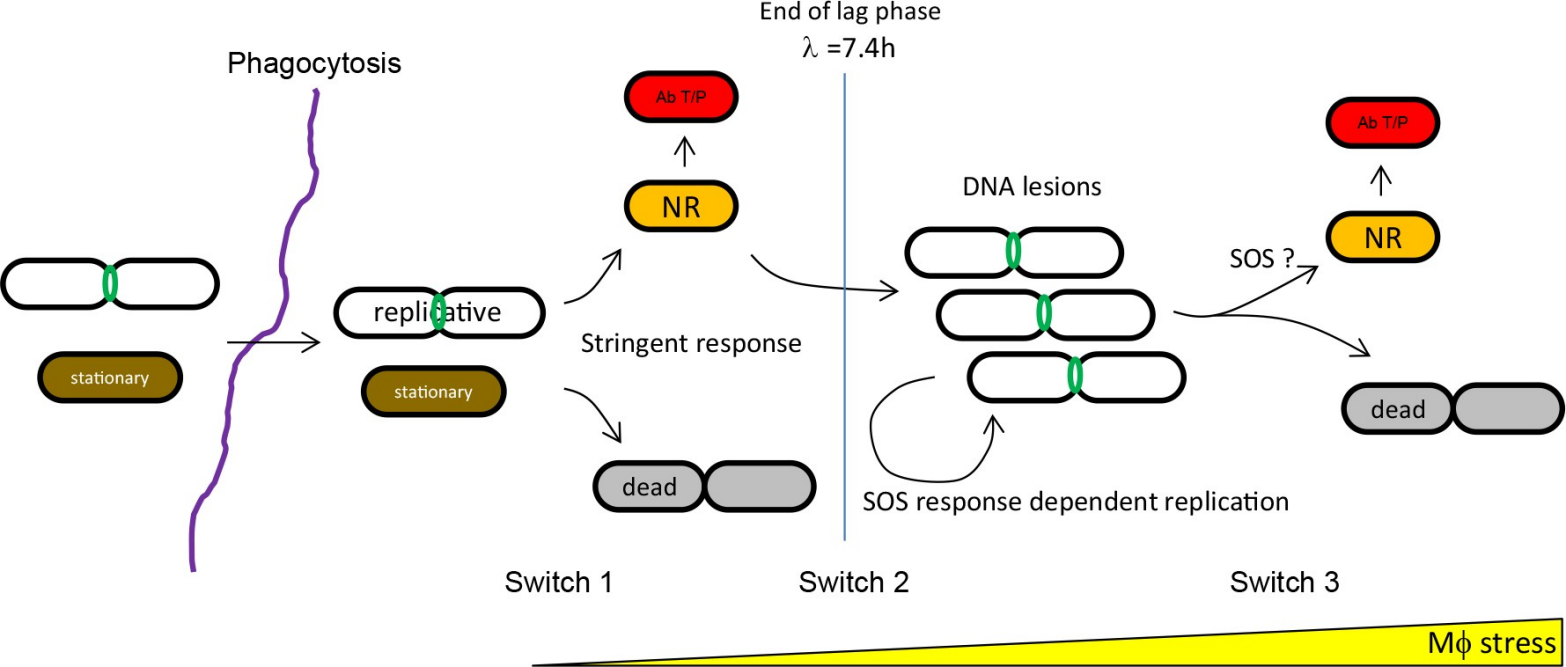
Dynamic of AIEC LF82 bacterial population during macrophage infection. Upon phagocytosis both replicative (green FtsZ ring) or stationary phase (brown) LF82 detect a signal, perhaps nutrient depletion, that led to stringent response activation. This activates a first phenotypic switch toward a non-replicating state (orange) that protects LF82 from dying because of initial stress burst. Among these non-replicating LF82 persisters are formed. After this lag phase, a second switch is required to initiate few rounds of replication. The timing and perhaps the frequency of switching from lag phase to replicative phase differentiate LF82 from our control commensal strain. We have not yet identified LF82 specific determinants that allow this switch. Subsequent replication rounds are dependent on the DNA repair machinery. A third switch, linked to the increasing stress or lesions, is turned on in a portion of the replicative population to form new non growers and persisters. The SOS response might also be playing a role at this stage.

We analyzed the growth and survival strategies used by the prototype AIEC strain LF82 to colonize the human monocytes-derived THP1 macrophages. Our analysis revealed that intracellular LF82 are constantly under stress while colonizing macrophages. The consequences of these stresses are important: increase in the death rate of bacteria, slow multiplication of replicating bacteria and formation of a large number of non-growing bacteria. LF82 adapts to this environment thanks to successive phenotypic switches that require the two main stress responses: the SOS response and the stringent response (Fig 7).

### Macrophages place LF82 under lethal stress

Using fluorescent reporters, we measured that half of the LF82 population present at 24 h P.I. had given rise to 6 or more generations. Under controlled laboratory conditions, this should produce a 30 to 60-fold increase in the population size at 20 h compared with the 1 h time point PI. However, we only observed a 3 to 6-fold increase in viable bacteria at 20–24 h compared with 1 h. We demonstrated that this modest colonization of macrophages by LF82 is explained by a big death rate and switch from replicative to non-growing cell cycle. At the single-macrophage and single-bacterium level, FD and TIMER fluorescent reporters revealed non-growing LF82. We observed macrophages containing few (less than 4) LF82 with red fluorescent dilution staining. These bacteria had therefore divided several times (>4) before observation and thus should be accompanied by their siblings (>16). This observation is in good agreement with our Live and Dead assay indicating that LF82 progeny has a significant chance to be killed and destroyed by the macrophage. By contrast, some macrophages contained growing LF82 and ultimately acquired more than 50 bacteria in one or several compartments. The live and dead assay confirmed that LF82 was frequently killed by macrophages. Because alterations of bacterial stress responses significantly reduced the bacterial yield, we propose that LF82 death is the consequence of oxidative, acid, genotoxic and protein stresses imposed by the macrophage. We compounded these experimental observations in a mathematical model describing the dynamics of bacterial infection within macrophages. A first phase of stalled growth, a likely combined effect of a prolonged lag phase and of compensation between death and division, is followed by an exponential increase in bacterial concentration. This second phase might correlate with a transient increased permissiveness of phagolysosomes or more likely the adaptation of LF82 to growth in this stressful environment. This expansion is successively curbed by the building-up of stressors. Many persisters are formed in the first phase. However, in the second phase a phenotypic switch to non-growing LF82 will eventually result in a sizeable increase of the persister population. We thus understand the survival of LF82 as a consequence of its ability to adapt to harsh phagolysosome environment both at the entry of the macrophage, by induction of stress responses and particularly the stringent response, and during exponential expansion by SOS response. LF82 advantage over K12-C600 would reside in its ability to exit from the lag phase to perform a few rounds of replication/division before stress becomes too strong (Fig 6). Strategically, early onset of growth is compensated by production of persistent bacteria, which endows the pathogenic strain with long-term survival in spite of rapid exploitation of the macrophage environment. We have not yet identified LF82 specific regulons, genes or mutations that allow this transition to take place.

### Replicative LF82

Fluorescent dilution revealed that after the exit of lag phase LF82 replicated moderately within macrophages, with generation time longer than 2 h. *In vitro*, this would be comparable with generation times observed in minimal medium with poor carbon sources such as acetate. Our observations revealed that within macrophages, 40–50% of the LF82 population presented an FtsZ ring, which is significantly above the number expected from a mixed population of *E*. *coli* growing with a 2h generation time (28% of cells with FtsZ ring [38]) and non-growing cells. Interestingly, in spite of SulA induction, we did not observe filamentation of LF82 within macrophage. These finding demonstrates that some of the cell cycle rules that were established under defined *in vitro* conditions do not apply to intracellular growth conditions, opening avenues for future investigations of bacterial cell cycle regulation in the context of host infection or the microbiota.

### Non-replicative LF82

FtsZ-GFP, FD and TIMER revealed that a significant number of intracellular LF82 were not growing. Using the three reporters allows to propose a scenario for this adaptation. FtsZ-GFP revealed that LF82 switch rapidly from a replicative mode before the infection to a non-dividing or slowly dividing mode within macrophage. This step is kept for the first hours of the infection. FD revealed that approximately 4% of the phagocytosed LF82 immediately halted their cell cycle. TIMER revealed that at 20 h P.I., approximately 10% of the LF82 population was not growing. We also demonstrated that once the macrophages were lysed, a large portion of the LF82 population (from 0.3 to 10%) was tolerant to several hours of antibiotic challenge, and the proportion of non-growers in the population increased in macrophages in the presence of antibiotics. Altogether, these observations suggest that the phagolysosome environment induces frequent cell cycle arrests among the population and that a part of this arrested population is persisters or tolerant to antibiotics. Such a phenomenon has been previously described during *S*. *typhimurium* infection of macrophages [16] or mice [16,36] and is reminiscent of VBNR mycobacteria [39]. Interestingly, we observed an increase in the proportion of macrophage-induced antibiotic-tolerant LF82 at later time points, suggesting adaptive responses to the intracellular microenvironment.

### Stress responses are important for LF82 survival within macrophages

As expected for bacteria residing in a toxic environment, stress responses are important for LF82 survival within macrophages. Acidic, oxidative, genotoxic, and envelope alterations, lack of Mg2+ and lack of nutrient stress responses significantly decreased the fitness of LF82 (50 to 10% of WT). In a few cases, we demonstrated an additive effect of simultaneously altering two pathways. However, LF82 demonstrated surprisingly good tolerance to these alterations compared with the *in vitro* findings for individual stresses. For example, *recA* deletion mutant was extremely sensitive (<1% survival) to prolonged treatment with genotoxic drugs (S3A Fig); by comparison, in macrophages, despite clear SOS induction, the viability of the *recA* mutant was only reduced by half compared with wild type bacteria. Set aside the possibility that stress-less niches exist due a possible heterogeneity in the macrophage population, the fitness decline of LF82 stress mutants may be limited by a combination of slow growth, formation of non-growers and/or yet uncharacterized adaptation pathways.

### Stringent and SOS responses successively control LF82 fate

We investigated the trigger that could allow some LF82 to halt their cell cycle inside macrophages. It appeared to be unrelated to the ability to sense acidic or oxidative stress. At an early time point, stringent response is required to block LF82 division (Fig 5D and 5E). Stringent response mutants show an altered survival and production of antibiotic-tolerant LF82 suggesting that abrupt nutrient starvation is one of the first signals received by LF82 upon phagocytosis. The early stringent response should result in a slowdown of transcription, translation and DNA replication, and therefore, it might provoke the formation of non-growers and a 7-h lag phase. This suggests that the slowing down induced by the stringent response confers a temporary protection than can be extended to antibiotic tolerance when bacteria become persisters. Accordingly the impact of stringent response alteration was less apparent after the lag phase when replication is re-established in a portion of the population of LF82. Our observations that stringent response is crucial to slow down replication and favor the formation of antibiotic tolerant persisters is in good agreement with recent data showing that inhibiting the stringent response blocks *Mycobacterium tuberculosis* entry into quiescence and reduces persistence [40] and the observation that DksA is a common mediator of persistence to various antibiotics [26]. SOS induction was moderate at 1 h but important at 6 h and 24 h P.I.; this is in good agreement with the lack of an effect of *recA* deletion on the accumulation of LF82 with FtsZ-GFP ring and with undiluted FD GFP. This is also in agreement with the lack of an influence of SOS mutants on the number of LF82 that were tolerant to cefotaxim at 1 h after infection. DNA lesions could, however, form during this period, but they were mostly observed when DNA replication and cell division restarted after 6 h. In this second phase of infection, SOS induction in replicative bacteria might play several roles: i) sustaining DNA repair and therefore DNA replication, cell division and increases in population size; ii) decelerating the division progression, this might be the role of SulA, and thus contributing to the formation of new non-growers; iii) intervening to help the return to growth of non-growers presenting DNA lesions, both within macrophages and after macrophage lysis.

### Macrophages as a niche for LF82 survival

The purpose of macrophage colonization by LF82 in Crohn’s disease patients is not yet understood. *In vitro*, LF82 colonization did not provoke extensive death of macrophages, which are thus unlikely to serve as a transient replicative niche for ileal infection. Alternatively, we can imagine that dormant LF82 within macrophages can serve as a long-term storage. In this environment, bacteria might be protected from competition with other species of the microbiota and coincidentally from antibiotics. Upon macrophage lysis or inactivation, dormant LF82 would be released and would start to multiply under adequate conditions.

## Methods

### Strains and plasmids

Deletion mutants (S1 Table) were constructed using the recombineering method as described in [41]. Plasmids are described in S2 Table.

### Infection and microscopy

THP1 (ATCC TIB-202) monocytes (5x10^5^ cells/ml) differentiated into macrophages for 18 h in phorbol 12-myristate 13-acetate (PMA, 20 ng/ml) were infected and imaged as previously described (Demarre *et al*., 2017). Infections were performed at MOI 30 (measured by CFU), resulting in the observation of 3 LF82 bacteria per macrophage on average at 1 h P.I.. Imaging was performed on an inverted Zeiss Axio Imager with a spinning disk CSU W1 (Yokogawa).

### Antibiotic challenge and viable bacterial count using the gentamycin protection assay

To determine the number of intracellular bacteria after 20 min of infection, infected macrophages were washed twice with PBS, and fresh cell culture medium containing 20 μg ml^−1^ of gentamicin (Gm) was added for the indicated time (1 h to 30 h). Cell monolayers were washed once with PBS, and 0.5 ml of 1% Triton X‐100 in 1x PBS was added to each well for 5 min to lyse eukaryotic cells [5]. Samples were mixed, diluted and plated on LB agar plates to determine the number of colony‐forming units (CFU) recovered from the lysed monolayers. For the antibiotic tolerance assays, macrophage lysates were transferred to 5-ml tubes and centrifuged for 10 min at 4100 g. The pellet was either suspended in 1x PBS (t0) and ciprofloxacin (1 μg/ml) for 1 h and 3 h, or in LB and cefotaxim (100 μg/ml) for 1 h and 3 h. CFU were measured by serial dilution. Tolerance was estimated for the 1-h and 3-h time points as a function of the CFU at t0. For intracellular tolerance assay ofloxacin (10 μg/ml) was added for 6h before imaging [37].

### Live and dead assay

At the indicated time points, macrophages were lysed with vigorous resuspension in 1x PBS 1% Triton. The cell lysate was pelleted at 300 g for 10 min to eliminate large cell remnants. The supernatant was centrifuged at 4000 g for 10 min. The bacterial pellet was suspended in 1x PBS and processed with the Live and Dead BacLight Viability kit (Thermo Fisher). The bacteria were pelleted for 3 min at 5000 g, resuspended in 50 μl of 1x PBS and spread on a 1% agarose 1x PBS pad for immediate observation.

### Measurement of gene expression by RT-qPCR

Total RNA was extracted with TRIzol reagent from 10^6^ macrophages infected for 1h, 6h or 24h or 10^7^ bacteria grown in cell culture medium for 3h as described in the *Molecular Cloning*, *a laboratory Manual* (Green and Sambrook, CSH Press). First-strand cDNA synthesis was performed with the Maxima First Strand cDNA Synthesis Kit for RT-qPCR (Thermo Fisher), and real-time qPCR was performed with SYBR Green Master Mix (Bio-Rad) on a MyiQ real-time qPCR machine (Bio-Rad).

### Fluorescence quantification

Custom-made FIJI macros were developed for the analyses of fluorescence. For the Biosensors constitutive expression of mCherry from p-mCherry was used to construct bacterial masks, which were subsequently used to measure GFP intensity. For TIMER analyses, green fluorescence was used to construct the mask. For biosensors and TIMER macrophages were lysed, bacteria were fixed and spread on agarose pad for the imaging. For FD, the analysis was directly performed on infected macrophages; the mean GFP fluorescence of mCherry objects was measured on single Z planes. To limit oversampling of bacteria in dense clusters, only two planes separated by >2 μm were analyzed per field of view. Fluorescence distributions were analyzed with the distribution fitting tool in MATLAB.

### Mathematical model for the infection kinetics

A set of three Ordinary Differential Equations (ODEs) recapitulates the main features of the observed growth within macrophages, as explained in the main text and in the SI:

$$\begin{array}{c} \frac{dB}{dt}=[1-I(t<\lambda )]\beta B-k_{p}B-I(t<\lambda )\delta_{1}B-\delta_{2}(S)B\\ \frac{dP}{dt}=k_{p}B\;(1)\\ \frac{dS}{dt}=B+P \end{array}$$

Here, *I*(*t*<*λ*) is the indicator function, which is unitary during lag phase. At the beginning of the infection, thus, the net growth rate -δ_1_ is either zero (K12-C600 and LF82) or negative (stringent response mutant LF82dskA). At later times, net growth rate β is instead positive. The stress-induced death rate has been chosen to be a sigmoidal function of the stress level:

$$\delta_{2}(S)=\frac{d_{max}}{1+e^{a(S-S_{1/2})}}$$

where the half-saturation stress value S_1/2_ and the sensitivity parameter *a* are assumed to be identical for all strains. Here stress is an effective variable quantifying the effect of crowding on growth within macrophages, and could correspond both to density-dependent reduction of bacterial growth rate (e.g. due to resource depletion), and to the progressive buildup of macrophage-induced killing.

### Fit of the infection kinetics data

Parameters providing the best fit of eqs. (1) to the times series of CFUs and persisters have been obtained by a weighted least-square distance minimization using the python differential evolution algorithm. We used a two-step approach to the fit which allowed us to establish first a subset of 7 parameters (λ and k_p_ for each strain and β) that shape the lag and exponential phases of growth. Subsequently, we fixed β and the λs, and fitted the remaining parameters. Details of the fitting procedure are found in the SI, and the results of the fit in S1 Text.

## Supporting information

S1 Fig Measure of the impact of various stresses on LF82 and K12-C600 growth. Growth curves of LF82 (green) and K12-C600 (blue) in LB medium at pH 7.4 and in the presence of serine hydroxamate (15 mg/ml), Serine hydroxamate (7 mg/ml), EDTA (70 mM), in LB at pH 4.7, LB at pH 4.7 in the presence of EDTA (70 mM), with addition of the antibiotics ciprofloxacin (24 ng/μl) or cefotaxim (800 ng/ml). Data are the mean of 3 technical replicates. Chemicals or a pH shift were applied at 160 min (red or green traces).
(PDF)

S2 Fig Viability and death assays. A) In situ Live and dead assay on infected macrophages (left panel). A very weak propidium iodide (PI) labeling is observed on putative dead LF82. By contrast strong PI labeling is observed after macrophage lysis (right panels). B) Measure of the speed of disappearance after phagocytosis by macrophages of heat-killed LF82. LF82 were killed by 15 min incubation at 60°C and subsequently labeled with propidium iodide. Labeled dead LF82 were incubated with macrophage at a MOI of 100. Imaging was performed at 1h, 2h, 3h and 24h post infection. SYTO-9 was used to reveal macrophage and eventual live bacteria. The number of dead LF82 per macrophage was measured at each time points. Data are average of 3 experiments. C) Measure of viable AIEC LF82 for 24 h post-infection of THP1 differentiated macrophages. Open circles represent individual infections, closed circles represent the median CFU (blue) ± Standard deviation (SD) (dotted lines).
(PDF)

S3 Fig Impact of SOS mutations on survival and persistence of LF82 strains. A) Measure of the resistance to Mitomycin C (MMC) of LF82, LF82 *recA*, LF82 *lexAind-*, MG1655, MG1655 *recA* and MG1655 *lexAind-*. B) Measure of the tolerance to cefotaxim of LF82 and LF82 *recA*, LF82 *lexAind-*, LF82 *sulA* grown in LB medium to an OD of 0.2. C) Induction of tolerance to cefotaxim by pretreatment with a subinhibitory dose of ciprofloxacin (24 ng/μl). The data represent the ratio of the number of bacteria that were tolerant to 3 h of cefotaxim in the presence or absence of ciprofloxacin.
(PDF)

S4 Fig LF82 regrowth after macrophage lysis. A) LF82 *Pasr-GFP*; B) LF82 *PkatG-GFP*. Macrophages were lysed 20h P.I. and immediately spread on LB agarose pad for imaging. Heat color scale represents the intensity of GFP fluorescence. Green and red arrows respectively indicate dividing and non-dividing bacteria.
(PDF)

S5 Fig Calibration of the fluorescent dilution assay. A) Growth curve of LF82 pFC6Gi in LB at 37°C. Colored diamonds represent the sampling times analyzed by fluorescence microscopy in the panel B. B) Distribution of GFP fluorescence in the growing population of LF82; GFP fluorescence is expressed as the number of generations (each generation corresponds to a 2-fold decrease in GFP fluorescence compared with the average fluorescence of the fully induced population at t0). C) Distribution of GFP fluorescence in the population of LF82-infecting macrophages. Fluorescence was measured for individual bacteria or small bacterial clusters on single Z planes after macrophage fixation at 1 h, 6 h, 24 h and 48 h post-infection (N = 500). D) Distribution of the GFP fluorescence in the population of K12-C600 bacteria infecting macrophages. Fluorescence was measured for individual bacteria or small bacterial clusters after macrophage fixation at 1 h and 24 h post-infection.
(PDF)

S6 Fig Calibration of the TIMER assay. A) Scatter plot of green versus red TIMER fluorescence measured for exponentially growing LF82 (green) and for a culture that had reached stationary phase (red). B) Distribution of the red TIMER fluorescence (Arbitrary fluorescence Intensity, AI) measured for exponentially growing LF82 (green) and stationary phase LF82 (red) at 4 h and 18 h post-infection. Curves represent the normal fit of the data. C) Inverse cumulative density function (CDF) of the TIMER red fluorescence distributions presented on the panel B and on Fig 5F. CDF allows to determine the number of fast replicating LF82 and non-growing LF82 for the WT, the *recA* and the *dksA* strains.
(PDF)

S1 Table Strains. (DOCX)

S2 Table Plasmids. (DOCX)

S1 Text Mathematical model for LF82 growth and survival within macrophages. (PDF)
