# Supplementary figures and images for: The Crohn’s disease-associated Escherichia coli strain LF82 relies on SOS and stringent responses to survive, multiply and tolerate antibiotics within macrophages
Source: PLoS Pathog. 2019 Nov 14;15(11):e1008123. doi: 10.1371/journal.ppat.1008123 (PMC6855411; doi:10.1371/journal.ppat.1008123)

A

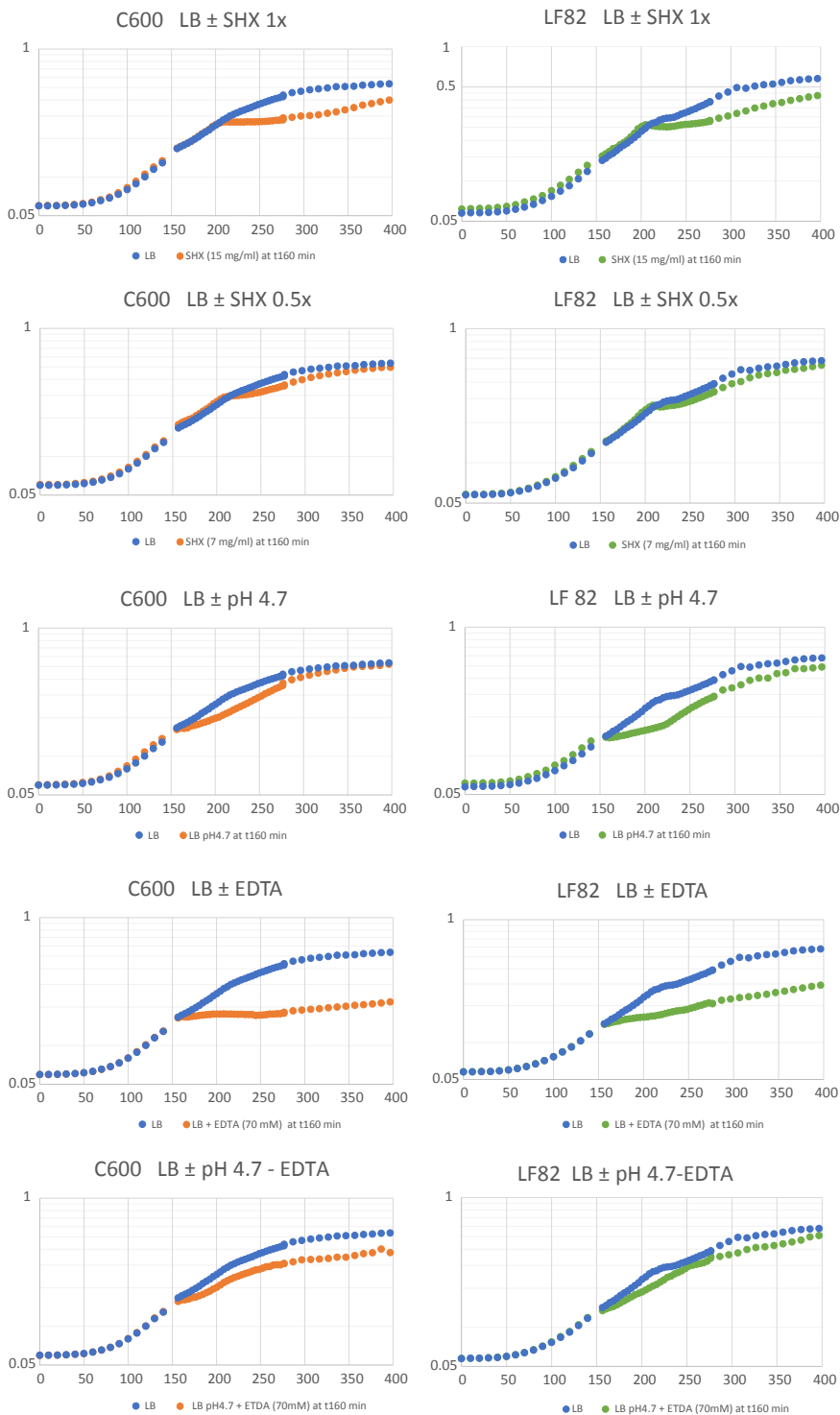

B

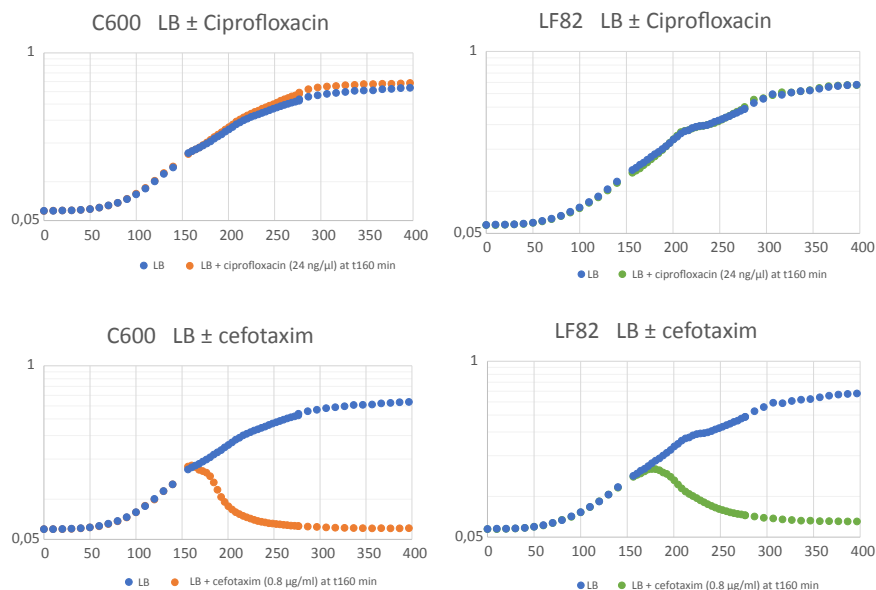

Supplement: S1 Fig — Growth curves of LF82 (green) and K12-C600 (blue) in LB medium at pH 7.4 and in the presence of serine hydroxamate (15 mg/ml), Serine hydroxamate (7 mg/ml), EDTA (70 mM), in LB at pH 4.7, LB at pH 4.7 in the presence of EDTA (70 mM), with addition of the antibiotics ciprofloxacin (24 ng/μl) or cefotaxim (800 ng/ml). Data are the mean of 3 technical replicates. Chemicals or a pH shift were applied at 160 min (red or green traces). (PDF) [file ppat.1008123.s001.pdf]

A

In situ live and dead labeling

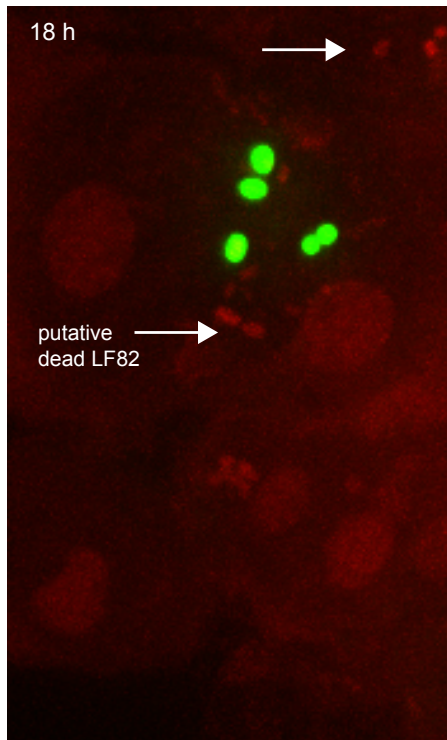

Live and dead labeling after macrophage lysis

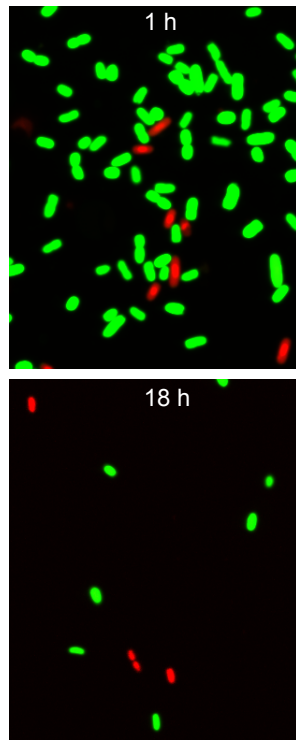

B

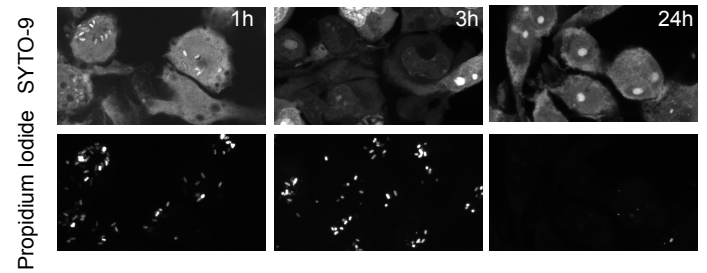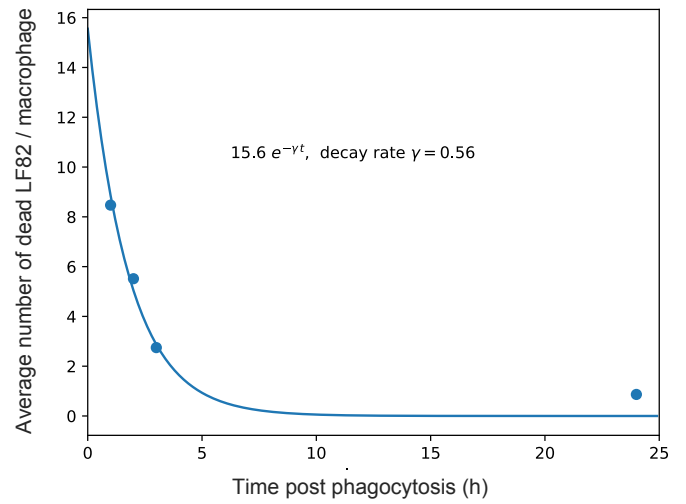

C

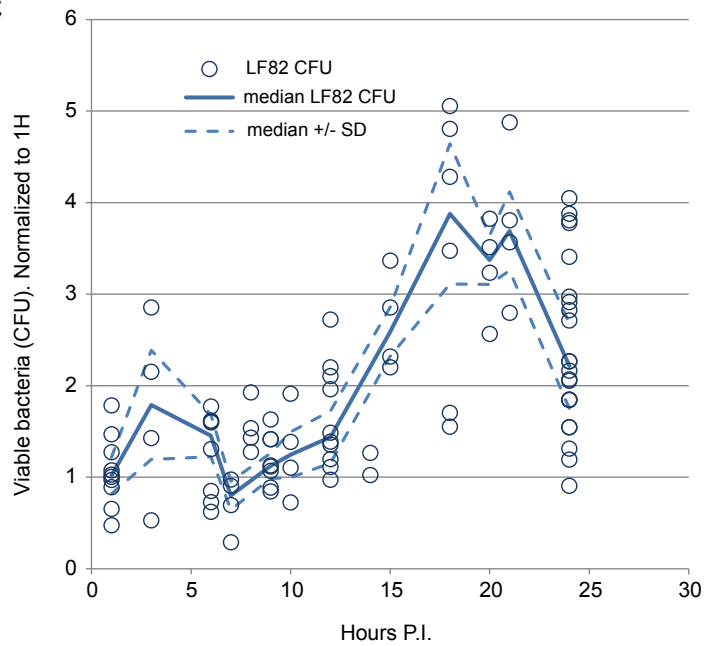

Supplement: S2 Fig — A) In situ Live and dead assay on infected macrophages (left panel). A very weak propidium iodide (PI) labeling is observed on putative dead LF82. By contrast strong PI labeling is observed after macrophage lysis (right panels). B) Measure of the speed of disappearance after phagocytosis by macrophages of heat-killed LF82. LF82 were killed by 15 min incubation at 60°C and subsequently labeled with propidium iodide. Labeled dead LF82 were incubated with macrophage at a MOI of 100. Imaging was performed at 1h, 2h, 3h and 24h post infection. SYTO-9 was used to reveal macrophage and eventual live bacteria. The number of dead LF82 per macrophage was measured at each time points. Data are average of 3 experiments. C) Measure of viable AIEC LF82 for 24 h post-infection of THP1 differentiated macrophages. Open circles represent individual infections, closed circles represent the median CFU (blue) ± Standard deviation (SD) (dotted lines). (PDF) [file ppat.1008123.s002.pdf]

A

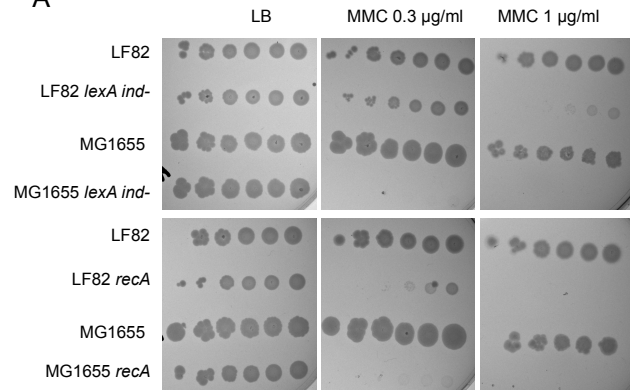

B

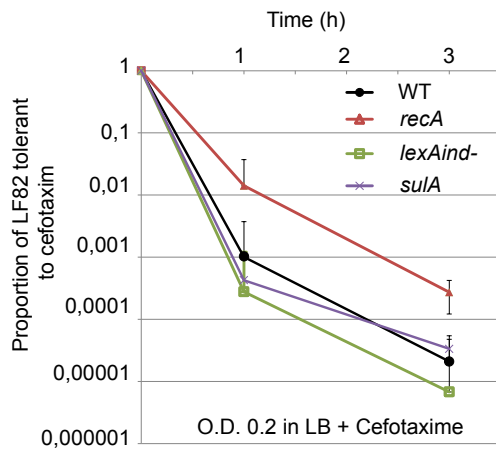

C

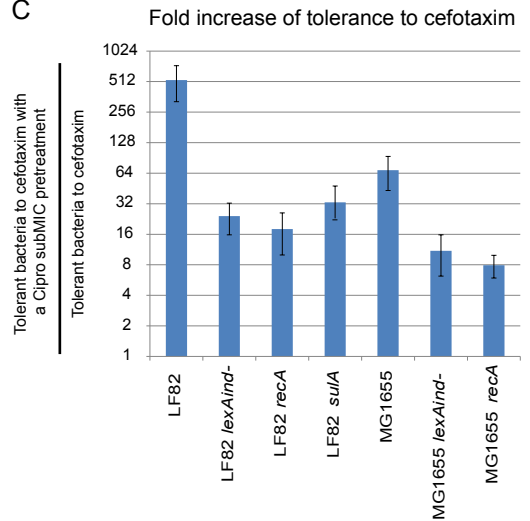

Supplement: S3 Fig — A) Measure of the resistance to Mitomycin C (MMC) of LF82, LF82 recA, LF82 lexAind-, MG1655, MG1655 recA and MG1655 lexAind-. B) Measure of the tolerance to cefotaxim of LF82 and LF82 recA, LF82 lexAind-, LF82 sulA grown in LB medium to an OD of 0.2. C) Induction of tolerance to cefotaxim by pretreatment with a subinhibitory dose of ciprofloxacin (24 ng/μl). The data represent the ratio of the number of bacteria that were tolerant to 3 h of cefotaxim in the presence or absence of ciprofloxacin. (PDF) [file ppat.1008123.s003.pdf]

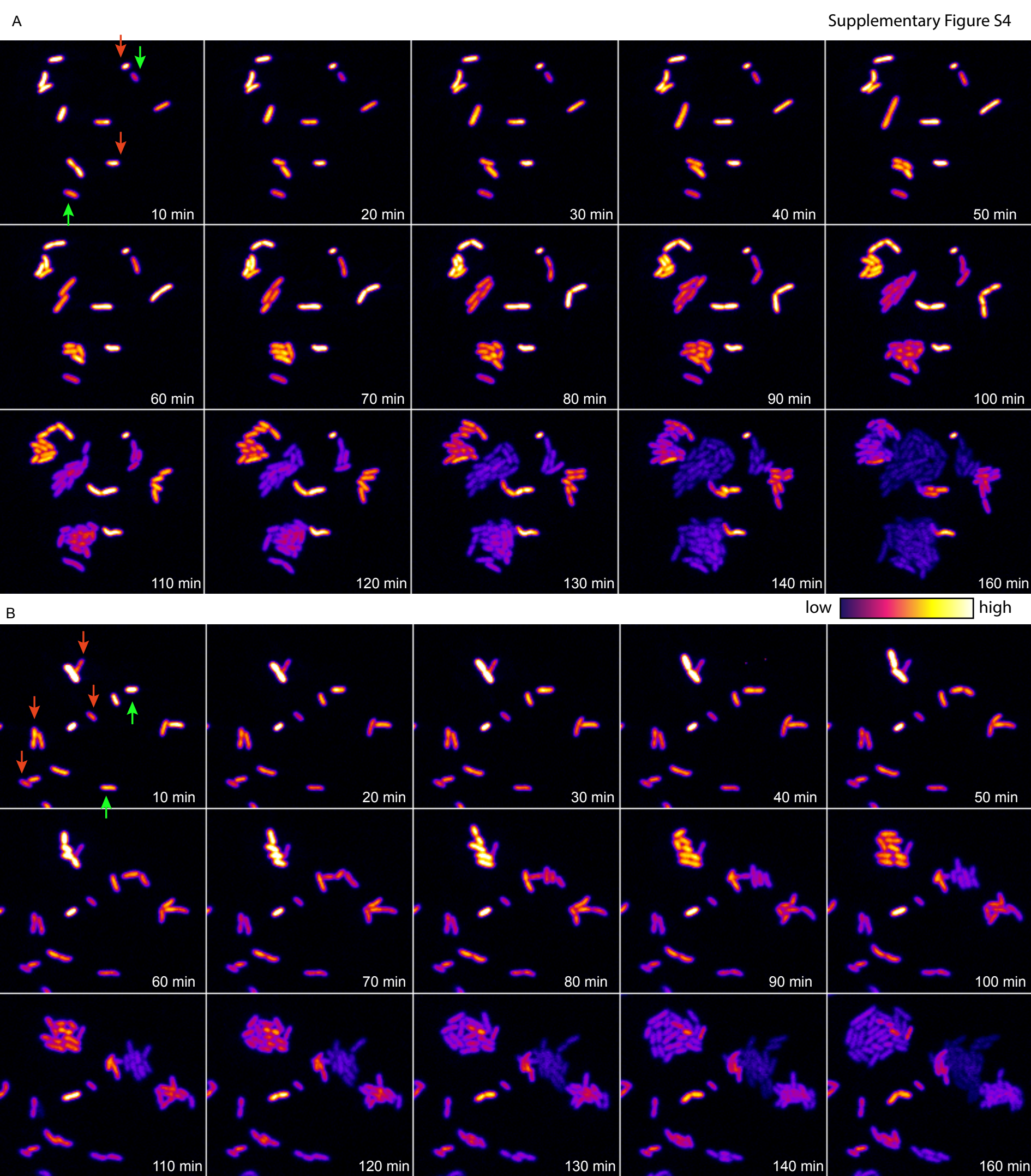

Supplement: S4 Fig — A) LF82 Pasr-GFP; B) LF82 PkatG-GFP. Macrophages were lysed 20h P.I. and immediately spread on LB agarose pad for imaging. Heat color scale represents the intensity of GFP fluorescence. Green and red arrows respectively indicate dividing and non-dividing bacteria. (PDF) [file ppat.1008123.s004.pdf]

A

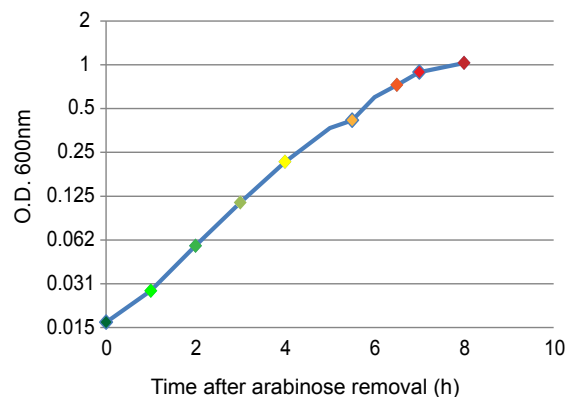

B

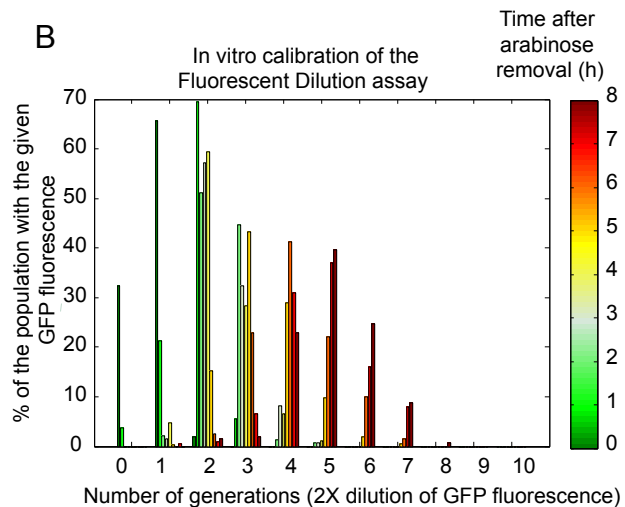

C

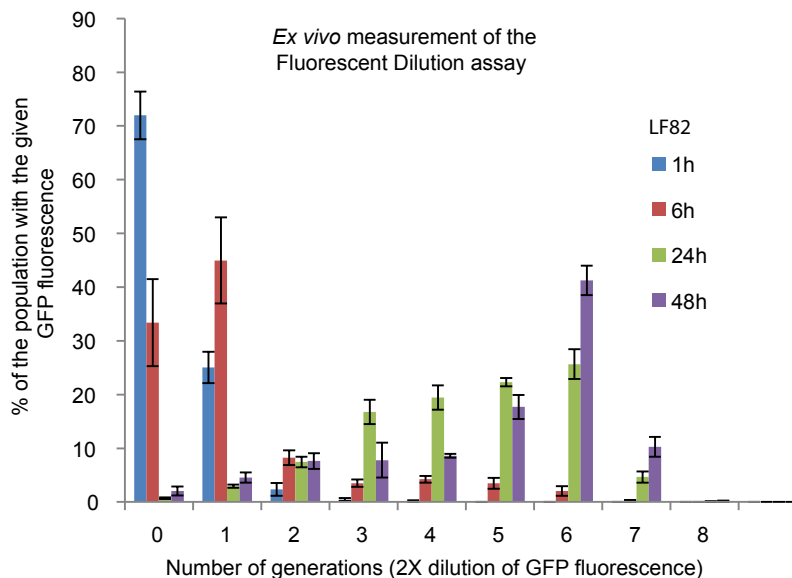

D

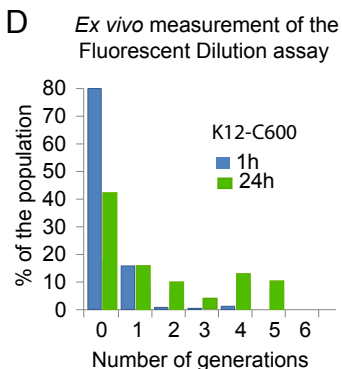

Supplement: S5 Fig — A) Growth curve of LF82 pFC6Gi in LB at 37°C. Colored diamonds represent the sampling times analyzed by fluorescence microscopy in the panel B. B) Distribution of GFP fluorescence in the growing population of LF82; GFP fluorescence is expressed as the number of generations (each generation corresponds to a 2-fold decrease in GFP fluorescence compared with the average fluorescence of the fully induced population at t0). C) Distribution of GFP fluorescence in the population of LF82-infecting macrophages. Fluorescence was measured for individual bacteria or small bacterial clusters on single Z planes after macrophage fixation at 1 h, 6 h, 24 h and 48 h post-infection (N = 500). D) Distribution of the GFP fluorescence in the population of K12-C600 bacteria infecting macrophages. Fluorescence was measured for individual bacteria or small bacterial clusters after macrophage fixation at 1 h and 24 h post-infection. (PDF) [file ppat.1008123.s005.pdf]

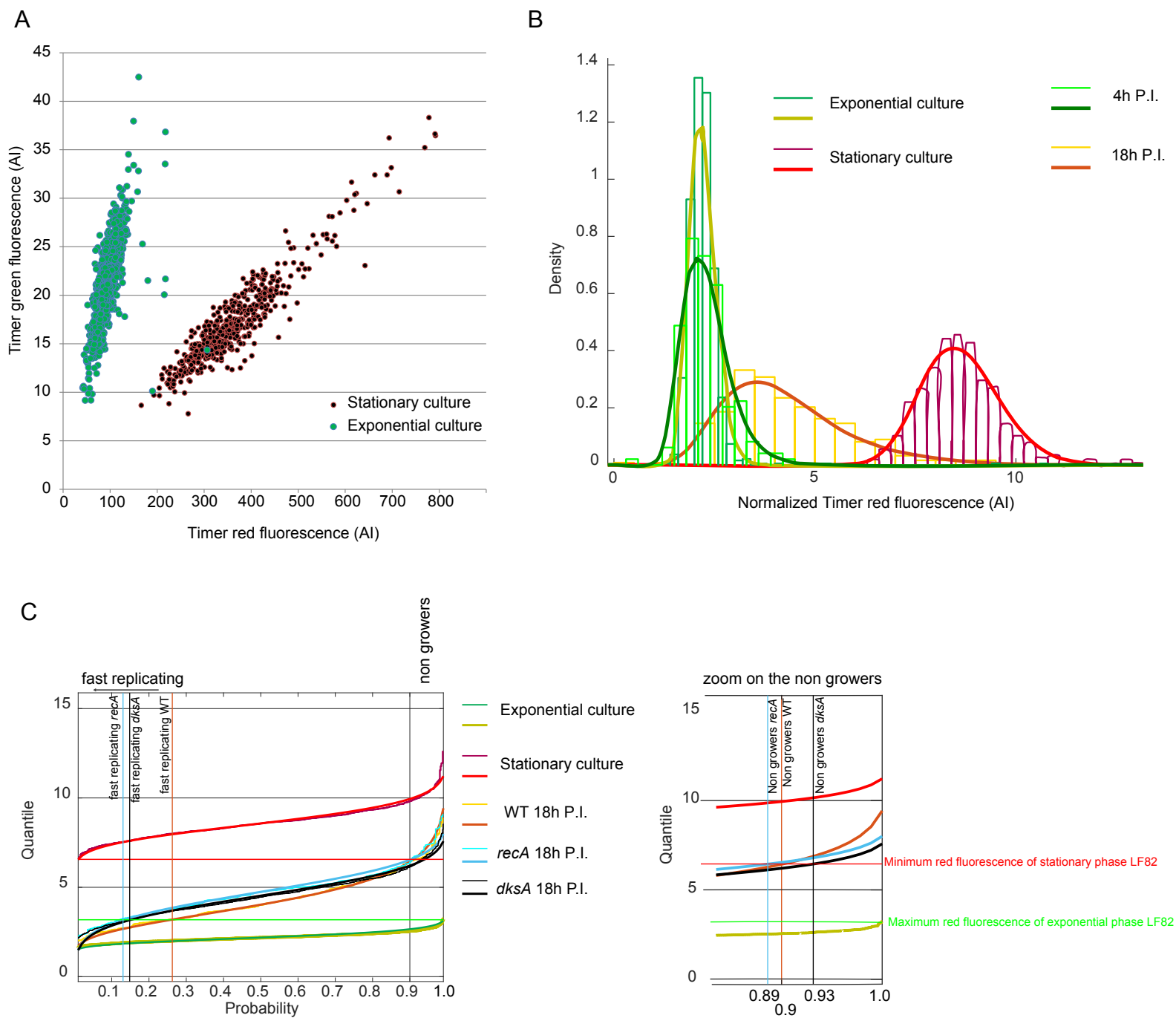

Supplement: S6 Fig — A) Scatter plot of green versus red TIMER fluorescence measured for exponentially growing LF82 (green) and for a culture that had reached stationary phase (red). B) Distribution of the red TIMER fluorescence (Arbitrary fluorescence Intensity, AI) measured for exponentially growing LF82 (green) and stationary phase LF82 (red) at 4 h and 18 h post-infection. Curves represent the normal fit of the data. C) Inverse cumulative density function (CDF) of the TIMER red fluorescence distributions presented on the panel B and on Fig 5F. CDF allows to determine the number of fast replicating LF82 and non-growing LF82 for the WT, the recA and the dksA strains. (PDF) [file ppat.1008123.s006.pdf]
