# Supplementary material for: The Crohn’s disease-associated Escherichia coli strain LF82 relies on SOS and stringent responses to survive, multiply and tolerate antibiotics within macrophages
Source: PLoS Pathog. 2019 Nov 14;15(11):e1008123. doi: 10.1371/journal.ppat.1008123 (PMC6855411; doi:10.1371/journal.ppat.1008123)
Supplement: S1 Table — (DOCX) [file ppat.1008123.s007.docx]

**S1 Table. Strains**

| Name | genotype | description | reference |
| --- | --- | --- | --- |
| AIEC LF82 |  |  | (Glasser *et al.,* 2001) |
| AIEC LF82 Δbla | *ampC* |  | Gift from Nicolas Barnich |
| AIEC LF82 Δ*htrA* | *htrA* |  | (Bringer *et al.,* 2005) |
| AIEC LFGD1 | AIEC LF82 *Δbla relA::kan* |  | This work |
| AIEC LFGD4 | AIEC LF82 *Δbla ydeO::kan* |  | This work |
| AIEC LFGD6 | AIEC LF82 *Δbla lon::kan* |  | This work |
| AIEC LFGD9 | AIEC LF82 *Δbla recA::kan* |  | This work |
| AIEC LFGD11 | AIEC LF82LF82 *Δbla HupA-mcherry-FRT-kan-FRT* |  | This work |
| AIEC LFGD13 | AIEC LF82 *Δbla pspA::kan* |  | This work |
| AIEC LFGD15 | AIEC LF82 *Δbla soxS::kan* |  | This work |
| AIEC LFGD27 | AIEC LF82 *Δbla phoP::kan* |  | This work |
| AIEC LFGD30 | AIEC LF82 *Δbla evgA evgS::kan* |  | This work |
| AIEC LFGD40 | AIEC LF82 *Δbla evgAS-FRT phoP::kan* |  | This work |
| AIEC LFGD41 | AIEC LF82 *Δbla evgAevgS-FRT ydeO::kan* |  | This work |
| AIEC LFGD56 | AIEC LF82 *Δbla relA-FRT* |  | This work |
| AIEC LFGD57 | AIEC LF82 *Δbla relA-FRT spoT::kan* |  | This work |
| AIEC LFGD69 | AIEC LF82 *Δbla lexAind- ::kan* | Mutation X-> Y in *lexA* constructed by recombineering | This work |
| AIEC LFGD79 | AIEC LF82 *Δbla dksA::kan* |  | This work |
| AIEC LFER1 | AIEC LF82 *Δbla lexAind-FRT dksA::kan* |  | This work |
| AIEC LFER2 | AIEC LF82 *Δbla dksA-FRT recA::kan* |  | This work |
| AIEC LFGD86 | AIEC LF82 *Δbla sulA::kan* |  | This work |
| AIEC LFGD83 | AIEC LF82 *Δbla ppk-ppX::kan* |  | This work |
| AIEC LF82 ΔrpoS | *rpoS::kan* |  | Gift from Jakob Moller Jensen (Simonsen *et al.,* 2011) |

1. Glasser AL, Boudeau J, Barnich N, Perruchot MH, Colombel JF, Darfeuille-Michaud A. Adherent invasive Escherichia coli strains from patients with Crohn’s disease survive and replicate within macrophages without inducing host cell death. Infect Immun. 2001;69: 5529–5537.

2. Bringer M-A, Barnich N, Glasser A-L, Bardot O, Darfeuille-Michaud A. HtrA stress protein is involved in intramacrophagic replication of adherent and invasive Escherichia coli strain LF82 isolated from a patient with Crohn’s disease. Infect Immun. 2005;73: 712–721. doi:10.1128/IAI.73.2.712-721.2005

3. Simonsen KT, Nielsen G, Bjerrum JV, Kruse T, Kallipolitis BH, Møller-Jensen J. A role for the RNA chaperone Hfq in controlling adherent-invasive Escherichia coli colonization and virulence. PloS One. 2011;6: e16387. doi:10.1371/journal.pone.0016387
