# Supplementary material for: The Crohn’s disease-associated Escherichia coli strain LF82 relies on SOS and stringent responses to survive, multiply and tolerate antibiotics within macrophages
Source: PLoS Pathog. 2019 Nov 14;15(11):e1008123. doi: 10.1371/journal.ppat.1008123 (PMC6855411; doi:10.1371/journal.ppat.1008123)
Supplement: S2 Table — (DOCX) [file ppat.1008123.s008.docx]

**S2 Table. Plasmids**

| name | description | Antibiotic resistance | reference |
| --- | --- | --- | --- |
| pKOBEGA |  | ampR specR | (Derbise *et al.,* 2003) |
| pAD37 | Matrix vector for recombineering | kanR | (David *et al.,* 2014) |
| pFWZ5 | Para-fts-sfGFP-T::aph | kanR | Gift from Fabai Wu  (Wu *et al.,* 2015) |
| pFCcGi | pFP25 P*rpsM*-*mCherry*, ParaBAD-GFP | ampR | Gift from Sophie Helaine  (Helaine *et al.,* 2014) |
| pP*rpsm*-*mcherry* | pGBM2-P*rpsM*-*mCherry* | specR | This work |
| pSC101-TIMER bac |  |  | Gift from Dirk Bumann (Claudi *et al.,* 2014) |
| pom1-GFP | pGBM2-Pro3-GFP |  | (Espéli *et al.,* 2001) |
| pLA42 | pFPV25 PkatG-gfpmut3 | ampR | Gift from Laurent Aussel (Viala *et al.,* 2011; Hébrard *et al.,* 2009) |
| pP1485 | pFPV25 P*asr-gfp* | ampR | Gift from Laurent Aussel (Viala *et al.,* 2011; Hébrard *et al.,* 2009) |
| pmgtC | pFPV25 P*mgtC-gfp* | ampR | Gift from Laurent Aussel (Viala *et al.,* 2011; Hébrard *et al.,* 2009) |
| pSulA-GFP | pZA31MCS-delta Xho P*sulA-GFP* |  | (Esnault *et al.,* 2007) |

1. Derbise A, Lesic B, Dacheux D, Ghigo JM, Carniel E. A rapid and simple method for inactivating chromosomal genes in Yersinia. FEMS Immunol Med Microbiol. 2003;38: 113–116. doi:10.1016/S0928-8244(03)00181-0

2. David A, Demarre G, Muresan L, Paly E, Barre F-X, Possoz C. The two Cis-acting sites, parS1 and oriC1, contribute to the longitudinal organisation of Vibrio cholerae chromosome I. PLoS Genet. 2014;10: e1004448. doi:10.1371/journal.pgen.1004448

3. Wu F, Van Rijn E, Van Schie BGC, Keymer JE, Dekker C. Multi-color imaging of the bacterial nucleoid and division proteins with blue, orange, and near-infrared fluorescent proteins. Front Microbiol. 2015;6: 607. doi:10.3389/fmicb.2015.00607

4. Helaine S, Cheverton AM, Watson KG, Faure LM, Matthews SA, Holden DW. Internalization of Salmonella by macrophages induces formation of nonreplicating persisters. Science. 2014;343: 204–208. doi:10.1126/science.1244705

5. Claudi B, Spröte P, Chirkova A, Personnic N, Zankl J, Schürmann N, et al. Phenotypic variation of Salmonella in host tissues delays eradication by antimicrobial chemotherapy. Cell. 2014;158: 722–733. doi:10.1016/j.cell.2014.06.045

6. Espéli O, Moulin L, Boccard F. Transcription attenuation associated with bacterial repetitive extragenic BIME elements. J Mol Biol. 2001;314: 375–386. doi:10.1006/jmbi.2001.5150

7. Viala JPM, Méresse S, Pocachard B, Guilhon A-A, Aussel L, Barras F. Sensing and adaptation to low pH mediated by inducible amino acid decarboxylases in Salmonella. PloS One. 2011;6: e22397. doi:10.1371/journal.pone.0022397

8. Hébrard M, Viala JPM, Méresse S, Barras F, Aussel L. Redundant hydrogen peroxide scavengers contribute to Salmonella virulence and oxidative stress resistance. J Bacteriol. 2009;191: 4605–4614. doi:10.1128/JB.00144-09

9. Esnault E, Valens M, Espéli O, Boccard F. Chromosome structuring limits genome plasticity in Escherichia coli. PLoS Genet. 2007;3: e226. doi:10.1371/journal.pgen.0030226
